# Perivascular cells induce microglial phagocytic states and synaptic engulfment via SPP1 in mouse models of Alzheimer’s disease

**DOI:** 10.1038/s41593-023-01257-z

**Published:** 2023-02-06

**Authors:** Sebastiaan De Schepper, Judy Z. Ge, Annerieke Sierksma, Gerard Crowley, Laís S. S. Ferreira, Dylan Garceau, Christina E. Toomey, Dimitra Sokolova, Javier Rueda-Carrasco, Sun-Hye Shin, Jung-Seok Kim, Thomas Childs, Tammaryn Lashley, Jemima J. Burden, Michael Sasner, Carlo Sala Frigerio, Steffen Jung, Soyon Hong

**Affiliations:** 1 UK Dementia Research Institute, Institute of Neurology, University College London, London, UK; 2 VIB-KU Leuven Center for Brain and Disease Research, Leuven, Belgium; 3 Leuven Brain Institute, KU Leuven, Leuven, Belgium; 4 The Jackson Laboratory, Bar Harbor, ME USA; 5 The Queen Square Brain Bank for Neurological Disorders, Department of Clinical and Movement Neuroscience, UCL Queen Square Institute of Neurology, London, UK; 6 Department of Clinical and Movement Neuroscience, UCL Queen Square Institute of Neurology, London, UK; 7 Department of Immunology and Regenerative Biology (IRB), Weizmann Institute of Science, Rehovot, Israel; 8 Department of Neurodegenerative diseases, UCL Queen Square Institute of Neurology, London, UK; 9 Laboratory for Molecular Cell Biology, University College London, London, UK

**Keywords:** Neuroimmunology, Alzheimer's disease, Mechanisms of disease, Molecular neuroscience

## Abstract

Alzheimer’s disease (AD) is characterized by synaptic loss, which can result from dysfunctional microglial phagocytosis and complement activation. However, what signals drive aberrant microglia-mediated engulfment of synapses in AD is unclear. Here we report that secreted phosphoprotein 1 (SPP1/osteopontin) is upregulated predominantly by perivascular macrophages and, to a lesser extent, by perivascular fibroblasts. Perivascular SPP1 is required for microglia to engulf synapses and upregulate phagocytic markers including *C1qa* and *Ctsb* in presence of amyloid-β oligomers. Absence of Spp1 expression in AD mouse models results in prevention of synaptic loss. Furthermore, single-cell RNA sequencing and putative cell–cell interaction analyses reveal that perivascular SPP1 induces microglial phagocytic states in the hippocampus of a mouse model of AD. Altogether, we suggest a functional role for SPP1 in perivascular cells-to-microglia crosstalk, whereby SPP1 modulates microglia-mediated synaptic engulfment in mouse models of AD.

## Abstract (web summary)

Microglia mediate aberrant synapse engulfment in Alzheimer’s disease (AD), but the underlying mechanisms are poorly understood. Here the authors show a perivascular cells-to-microglia crosstalk that induces microglia phagocytic state resulting in synapse engulfment in two mouse models of AD.

## Main

The integrity of neurons and synapses is critically dependent on brain-resident macrophages, which include microglia and perivascular macrophages (PVMs) that continuously monitor and clear phagocytic targets across the lifespan^1,2^. Microglia, tissue-resident macrophages of brain parenchyma, contribute to circuit refinement through engulfment of synapses and axon tracts during postnatal development, whereas PVMs associate with the perivascular space where they represent the first responders to toxic agents and pathogens that may cross the blood–brain barrier (BBB) into the brain parenchyma^3–7^. Microglia and PVMs both share similar yolk-sac origin and reside on the parenchymal side of the BBB, yet occupy distinct microenvironments and express unique cell-type-specific markers^8–13^. Because of the immediate juxtaposition of perivascular space with the brain parenchyma, we reasoned that microglia and PVMs could potentially influence each other to coordinate phagocytosis in response to central nervous system perturbations. For example, in Alzheimer’s disease (AD), many of the identified risk variants point toward defective phagocytic and endolysosomal pathways in microglia and PVMs; however, intercellular and intracellular mechanisms governing this impairment remain unclear^14^. In line, microglia mediate regional synapse loss in AD, but environmental cues that modulate potential microglial functional states are poorly understood^15^.

Here we uncovered secreted phosphoprotein 1 (SPP1/osteopontin), predominantly derived from PVM, as an extrinsic modulator of microglial phagocytosis. We observed that SPP1 is required for activation of complement-initiator C1q and synaptic engulfment by microglia in AD mouse models. SPP1 has been shown in multiple peripheral tissues to regulate phagocytosis by macrophages^16–20^. In AD patients, secreted SPP1 levels are increased in cerebrospinal fluid (CSF) and plasma; further, SPP1 is found on plaque-associated microglia and has been suggested as a conserved disease-associated marker among mice and human^21–27^. However, the role of SPP1 in the brain or its relevance in AD is unknown. Here we found a region-specific activation of SPP1 in the hippocampal perivascular space of AD mouse models as well as in AD patient tissues. Super- and ultra-structural examination revealed that SPP1 is expressed predominantly by PVMs, and to a lesser extent, by perivascular fibroblasts (PVFs) in the adult hippocampus. In mouse models of AD, we found that perivascular SPP1 is upregulated in a region-specific manner at onset of synaptic elimination by microglia. Genetic ablation of SPP1 ameliorated microglial phagocytic states in AD mouse models as well as C1q activation, leading to the prevention of microglia–synapse engulfment and synapse loss in these mice despite amyloid-β (Aβ) challenge. Using single-cell RNA sequencing (scRNA-seq) and computational ligand–target predictions (NicheNet^28^), we noted multiple autocrine and paracrine signaling pathways to be potentially modulated by perivascular SPP1, with many of these pathways converging on microglial phagocytic functional states. Altogether, our data suggest a functional crosstalk between PVMs and microglia and propose perivascular SPP1 as an extrinsic signal that modulates synapse phagocytosis by microglia.

## Results

### SPP1 upregulation at onset of microglia–synapse phagocytosis

**Fig. 1:**
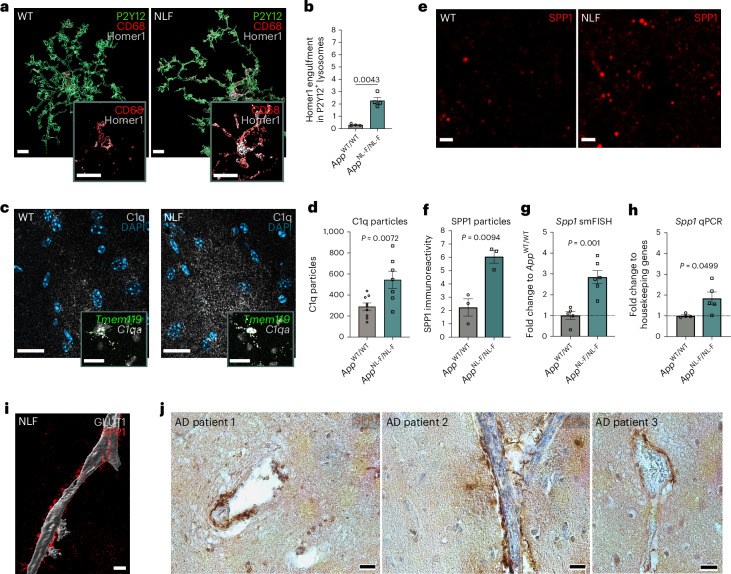
SPP1 upregulation at onset of microglia–synapse phagocytosis. **a**, Representative 3D reconstructed images showing Homer1 engulfment within CD68^+^ lysosomes of P2Y12^+^ microglia in 6-month *App*^WT^ (WT) versus *App*^NL-F^ CA1 hippocampal SLM. Scale bar represents 5 µm. **b**, Quantification of Homer1 engulfment in 6-month WT and *App*^NL-F^ P2Y12^+^ microglia. One datapoint represents average of 1 mouse from *n* = 4 animals per genotype examined over two independent experiments. *P* values from two-tailed unpaired Student’s *t*-test with Welch’s correction. **c**, Representative confocal images of C1q protein expression in 6-month WT versus *App*^NL-F^ mice. Insert represents *C1qa* mRNA within *Tmem119*^*+*^ microglia. Scale bar represents 20 µm. Data are representative of two mice per genotype examined over at least five independent experiments. **d**, Quantification of C1q puncta in 6-month WT and *App*^NL-F^ CA1 hippocampus. One datapoint represents one ROI per mouse from *n* = 9 WT mice and *n* = 7 *App*^NL-F^ mice examined over two independent experiments. Average amount of cells per datapoint is 30–40. *P* values from two-tailed unpaired Student’s *t*-test. **e**,**f**, 3D-τ-STED imaging of secreted SPP1 in 6-month WT and *App*^NL-F^ SLM (**e**) and quantification of SPP1 total fluorescence particles (**f**). One datapoint represents one ROI per mouse, with total of *n* = 3 mice examined over one independent experiment. *P* values from two-tailed unpaired Student’s *t*-test. Scale bar represents 2 µm. **g**,**h**, Quantification of *Spp1* expression within hippocampus of 6-month WT versus *App*^NL-F^ as measured by smFISH in hippocampus (**g**) or via qPCR on hippocampal homogenates (**h**). One datapoint represents one individual value per mouse, with total of *n* = 5–6 mice (per genotype) (**g**) or 4–5 mice (per genotype) (**h**) examined over two independent experiments. *P* values from two-tailed unpaired Student’s *t*-test. **i**, Representative 3D reconstruction of SPP1 adjacent to GLUT1^+^ vasculature in SLM of 6-month *App*^NL-F^. Scale bar represents 10 µm. Image representative of two *App*^NL-F^ mice examined over four independent experiments. **j**, Representative images of SPP1 expression along vasculature in postmortem hippocampal brain slices of three AD patients. Images are representative of six AD patients (see also Supplementary Table 1). Scale bar represents 20 µm. Data are shown as mean ± s.e.m. Source data

**Extended Data Fig. 1:**
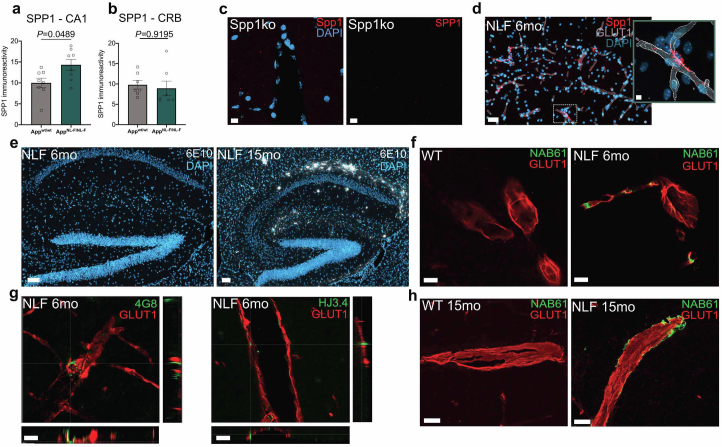
Pre-plaque vascular Aβ deposition in *App*^NL-F^ mice. **(a**, **b)** Quantification of SPP1 immunoreactivity in CA1 hippocampus **(a)** versus cerebellum as control region **(b)**, as measured by confocal imaging. 1 datapoint represents 1 ROI per mouse hippocampus, with total of 8 (CA1, **a**) and 7 (CRB, **b**) mice per genotype examined over 2 independent experiments. *P* Values from linear mixed-effect model with multivariate t-distribution post-hoc test. Data are shown as Mean ± SEM. **(c)** Validation of SPP1 antibody (left) and anti-*Spp1* probe for smFISH (right) in 3 mo *Spp1*^*KO/KO*^ hippocampal tissue. Scale bar represents 10 µm. Data representative of n = 4 *Spp1*^*KO/KO*^ mice examined over 1 independent experiment. **(d)** Representative confocal image of *Spp1* mRNA expression along GLUT1^+^ vasculature in SLM of 6 mo *App*^NL-F^ mice as characterized by smFISH-IHC. Scale bar represents 20 µm. Insert: 3D reconstruction. Scale bar represents 5 µm. Data are representative of 3 *App*^NL-F^ mice examined over at least 4 independent experiments. **(e)** Representative confocal images showing 6E10 plaque staining in hippocampus of 6 mo versus 15 mo *App*^NL-F^ mice. Scale bar represents 100 µm. Data representative of n = 3 *App*^NL-F^ mice mice per time point, examined over 2 independent experiments. **(f)** Representative confocal images showing oAβ around GLUT1^+^ blood vessels as characterized by NAB61 immunoreactivity in 6 mo WT versus 6 mo *App*^NL-F^ SLM. Scale bar represents 10 µm. Data representative of n = 3 mice per genotype, examined over 2 independent experiments. **(g)** Representative confocal images showing vascular oAβ deposition using alternative antibodies 4G8 and HJ3.4 that recognize Aβ17-24 and Aβ1-13, respectively, in 6 mo *App*^NL-F^ SLM. Scale bar represents 10 µm. Data representative of n = 3 *App*^NL-F^ mice examined over 2 independent experiments. **(h)** Representative confocal images showing vascular oAβ (NAB61) in 15 mo WT versus *App*^NL-F^ SLM. Scale bar represents 20 µm. Source data

To address whether SPP1 is dysregulated at a time point when synapses are vulnerable to microglial engulfment in an AD-relevant context, we used the slow-progressing *App*^NL-F^ mouse model, where control by the endogenous *App* promoter allows for physiological cell-type specific and temporal regulation of Aβ production^15,29,30^. We first assessed phagocytic microglia–synapse interactions in the *App*^NL-F^ hippocampus at the age of 6 months, an age that precedes robust plaque deposition in the hippocampal parenchyma^30^. We quantified internalized level of Homer1-immunoreactive synaptic puncta within CD68^+^ P2Y12^+^ microglial lysosomes and observed an approximate sevenfold increase in synaptic engulfment by microglia in *App*^NL-F^ mice as compared to those of age- and sex-matched wild-type (WT) mice (Fig. 1a,b). We also observed upregulation of C1q, the initiating protein of the classical complement cascade that mediates microglial phagocytosis of synapses^3,15,31^ (Fig. 1c,d). *C1qa* expression was contained within *Tmem119*^+^ microglia as assessed by single-molecule fluorescent in situ hybridization (smFISH), confirming microglia as the main producers of C1q in the adult hippocampus (Fig. 1c, insert)^15,32^. We next assessed potential cross-regulation between synaptic engulfment and SPP1, a glycoprotein associated with phagocytosis in peripheral macrophages, in the 6-month *App*^NL-F^ hippocampus^18^. Using 3D-stimulated emission depletion (3D-τ-STED) super-resolution imaging, we found an approximately threefold increase of punctate SPP1 protein immunoreactivity in the CA1 hippocampus of *App*^NL-F^ mice as compared to age- and sex-matched WT controls (Fig. 1e,f). The SPP1 upregulation was region-specific, that is, in hippocampus but not cerebellum (Extended Data Fig. 1a,b). In line with increased SPP1 production, we found an approximate threefold increase of *Spp1* mRNA expression levels in hippocampal CA1 sections of *App*^NL-F^ mice compared to WT mice by smFISH in intact tissue, which was further confirmed by qPCR analysis on brain homogenates (Fig. 1g,h). The specificity of SPP1 antibody and *Spp1-*targeting smFISH probes was validated by absence of signals in *Spp1*^*KO/KO*^ mice (Extended Data Fig. 1c).

Interestingly, *Spp1* mRNA expression in the hippocampal CA1 was specifically enriched within the stratum lacunosum-moleculare (SLM) layer and *Spp1*^*+*^ cells displayed an elongated vascular-like pattern (Extended Data Fig. 1d). Costaining with pan-endothelial marker GLUT1 showed cellular zonation of *Spp1* mRNA expression adjacent to vascular structures, whereas outside of the endothelial membrane, no *Spp1* mRNA was detected (Extended Data Fig. 1d, inset). Using high-resolution confocal imaging and 3D surface rendering, we confirmed the close association of cytosolic SPP1 protein expression with GLUT1^+^ vasculature (Fig. 1i). Similar to the murine *App*^NL-F^ hippocampus, we also found a striking presence of SPP1 immunoreactivity along the vasculature in the hippocampus of postmortem AD patient tissues (Fig. 1j and Supplementary Table 1). Altogether, these data suggest that SPP1 expression is enriched along the vasculature in *App*^NL-F^ mice and AD patients. Further, we found negligible levels of Aβ oligomers (oAβ) or plaques at this early time point in the parenchyma of *App*^NL-F^ hippocampus (Extended Data Fig. 1e)^30^. In contrast, however, we observed oAβ deposition along the hippocampal vasculature at 6 months, assessed by the oAβ-specific NAB61^+^ immunostaining juxtaposed to GLUT1^+^ vasculature (Extended Data Fig. 1f)^33^. The latter was confirmed using two other anti-Aβ antibodies, the 4G8 and HJ3.4, which recognize Aβ17-24 and Aβ1-13, respectively (Extended Data Fig. 1g)^34^. The vascular oAβ deposition was more pronounced in 15-month *App*^NL-F^ mice, in contrast to age-matched WT controls in which no positive NAB61 staining was found (Extended Data Fig. 1h). These data suggest early, preplaque vascular Aβ deposition in the *App*^NL-F^, coinciding with SPP1 activation in the perivascular space.

### SPP1 is expressed by PVMs and fibroblasts

**Fig. 2:**
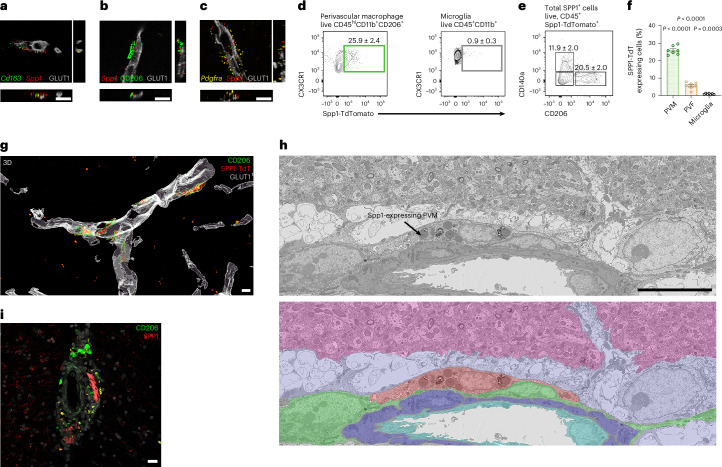
SPP1 is expressed by PVMs and fibroblasts. **a**–**c**, Representative images of *Spp1* mRNA expression juxtaposed to GLUT1^+^ vasculature, colocalizing with pan-PVM markers *Cd163* (**a**), CD206 (**b**) and PVF (*Pdgfra*^+^) (**c**) in 6-month *App*^NL-F^ SLM as characterized by smFISH-IHC. Scale bar represents 10 µm. Data are representative of four *App*^NL-F^ mice examined over two independent experiments. **d**–**f**, Representative FACS plots to identify PVMs (CX3CR1^+^CD45^+^CD11b^+^CD206^+^ (**d**), microglia CX3CR1^high^CD45^+^CD11b^+^ (**e**) or gated on total TdT expressing cells isolated from *Spp1*^TdT^ hippocampal homogenates and quantification (**f**)). One datapoint represents one cell type per mouse (PVM, PVF and microglia) pooled from *n* = 7 mice examined over two independent experiments (**f**). *P* values from one-way ANOVA, Bonferroni’s multiple comparison test. **g**, Three-dimensional reconstruction of CD206^+^ PVMs expressing SPP1-Td along GLUT1^+^ vessels in SLM from *Spp1*^TdT^ mice. Scale bar represents 7 µm. Data are representative of three *Spp1*^TdT^ mice examined over two independent experiments. **h**, Representative single serial section SEM backscatter electron image of a representative SPP1-TdT-positive PVM as identified by CLEM (Upper). SPP1-TdT-positive cell manually pseudocolored red, together with neuropil (pink), astrocytes (lilac), smooth muscle cells (purple), endothelial cells (cyan) and other perivascular cells (green), shown with reduced opacity over the electron microscopy data (lower). Accompanying confocal overlays and correlation images shown in Extended Data Fig. 3e,d array tomography data shown in Supplementary Video 1. Scale bar represents 10 µm. **i**, Representative image of perivascular SPP1 in AD postmortem hippocampal tissue, costained with CD206. Scale bar represents 25 µm. Data are representative of *n* = 6, six different patient tissues (Supplementary Table 1). Data are shown as mean ± s.e.m. TdT, TdTomato. Source data

**Extended Data Fig. 2:**
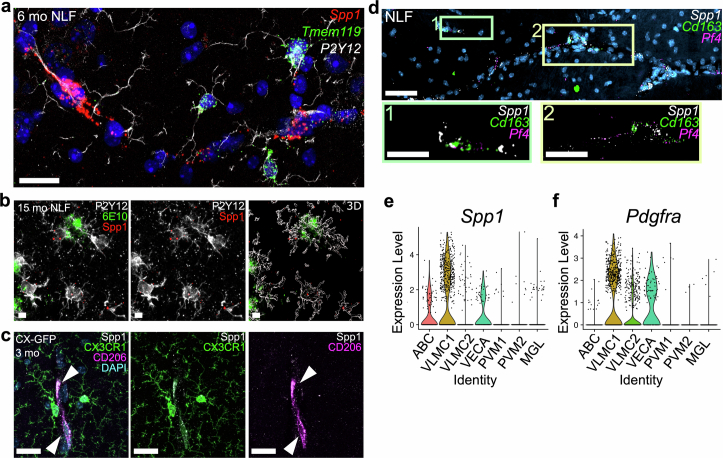
*Spp1* is expressed in perivascular cells. **(a)** Representative confocal image showing *Spp1* and *Tmem119* mRNA expression within P2Y12^+^ microglia of 6 mo *App*^NL-F^ SLM as characterized by smFISH-IHC. Scale bar represents 20 µm. Data representative of n = 8 *App*^NL-F^ mice examined over 3 independent experiments. **(b)** Representative confocal image showing *Spp1* mRNA in P2Y12 microglia associated with 6E10^+^ plaques in 15 mo *App*^NL-F^ SLM. Scale bar represents 5 µm. Data representative of n = 3 *App*^NL-F^ mice examined over 2 independent experiments. **(c)** Representative confocal images of SPP1 protein expression in CD206^+^ PVMs (arrow head) and CX3CR1^GFP^ myeloid cells of 3 mo *Cx3cr1*^*GFP/WT*^ SLM^86^. Scale bar represents 20 µm. Data are representative of n = 2 *Cx3cr1*^*GFP/WT*^ mice examined over at least 3 independent experiments. **(d)** Representative confocal image showing *Spp1* mRNA expression occasionally colocalizing with pan-PVM markers *Cd163* and *Pf4 in* 6 mo *App*^NL-F^ SLM as identified by smFISH. Scale bar represents 50 µm. Data representative of n = 6 *App*^NL-F^ mice examined over 2 independent experiments. **(e**, **f)** Violin plots of *Spp1* **(e)** and *Pdgfra* **(f)** expression reanalyzed from Zeisel et al.^37^. VLMC, vascular leptomeningeal cells; ABC, Arachnoid barrier cells; VECA, Arterial vascular endothelial cells; PVM, Perivascular macrophage; MGL, Microglia.

Spp1 expression in 6-month *App*^NL-F^ hippocampus was remarkably restricted to cells along the perivascular space (Fig. 1). Given the role of SPP1 as a marker of macrophage subsets in peripheral tissues, we assessed whether Spp1 is expressed by brain-resident macrophages associated with the vascular space, that is *Cd163*^*+*^ CD206^+^ PVM^35^. *Spp1* mRNA expression predominantly colocalized with pan-PVM markers *Cd163* and CD206 juxtaposed to GLUT1^+^ hippocampal vasculature (Fig. 2a,b). In contrast, we failed to observe *Spp1* expression in *Tmem119*^hi^ P2Y12^+^ microglia of 6-month *App*^NL-F^ animals (Extended Data Fig. 2a). Of note, *Spp1* expression in microglia was detected only at later stages, that is, in 6E10^+^ plaque-rich 15-month *App*^NL-F^ hippocampus (Extended Data Fig. 2b), likely reflecting SPP1^+^ disease-associated microglia^25,36^. Further, immunophenotypic characterization revealed restricted expression of SPP1 within CD206^+^CX3CR1^+^ cells, and *Spp1*^+^ cells were found positive for PVM-specific platelet factor 4 (*Pf4*) and *Cd163* (Extended Data Fig. 2c,d)^11,13,37^. Interestingly, we also found rare *Spp1*^*+*^ *Cd163*^*−*^ *Pf4*^*−*^ cells in the hippocampal perivascular space (Extended Data Fig. 2d). *Spp1* mRNA has been suggested by scRNA-seq to be expressed by vascular leptomeningeal cells, also known as PVFs, which are located within parenchymal arteries (Extended Data Fig. 2e)^37^. Indeed, the second cluster of *Spp1*^*+*^ cells coexpressed the pan-PVF marker *Pdgfra* (encoding CD140a), suggesting PVFs as a second cellular source of SPP1 surrounding GLUT1^+^ vasculature in the hippocampus, albeit much less abundant compared to PVMs (Extended Data Fig. 2f,c).

**Extended Data Fig. 3:**
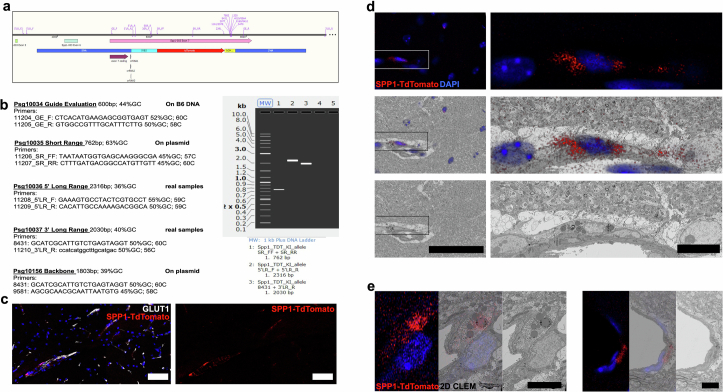
*Spp1*^TdT^ reporter mouse development. **(a)** Design of the knock-in allele is shown, with an IRES and TdTomato (TdT) reporter inserted at the stop codon in exon 7 in the mouse *Spp1* locus. Top, the mouse locus with locations of primers used for validation of the inserted construct and later genotyping for the reporter construct is shown. Below, schematic displays the upstream (5’HA) and downstream (3’HA) homology arms in blue; IRES in aqua; TdT reporter gene in red; and bovine growth hormone poly(A) region in yellow. **(b)** PCR primers used to confirm incorporation of construct are listed, with primer names based on genomic locations as shown in B. Gel shows appropriate size bands for incorporation of plasmid construct (lane 1), for left arm integration event (lane 2) and right arm integration event (lane 3). Primer pairs for plasmid backbone alone are negative (lanes 4 and 5). **(c)** Representative confocal image showing SPP1-TdT expression along GLUT1^+^ vessels in hippocampus of 3 mo *Spp1*^TdT^ reporter mice. **(d)** Left panels show low magnification overview of SPP1-TdT^+^ cell (red), and all nuclei (blue) in the hippocampus, as shown in Fig. 2h, scale bar 50 µm. Top, maximum intensity projection of confocal stack; middle, maximum intensity projection shown at reduced opacity over the correlated back scattered electron image of serial section 79 of the same region of the CLEM sample; bottom, back scattered electron image of serial section 79 of the same region of the CLEM sample alone. Box highlights region acquired across 200 serial sections. Right panels show boxed region at higher magnification with serial section 85. Scale bar represents 10 µm. Data representative of n = 4 *Spp1*^TdT^ mice examined over 2 independent experiments. **(e)** Two additional CLEM examples of SPP1-TdT^+^ cells targeted and correlated with volume electron microscopy. Left panels show maximum intensity projection of confocal stack; middle panels, maximum intensity projection shown at reduced opacity over the correlated back scattered electron image of a central serial section of the same region of the CLEM sample; right panels, back scattered electron image of the same region of the CLEM sample alone. Scale bar represent 5 µm. Data representative of n = 4 *Spp1*^TdT^ mice examined over 2 independent experiments.

To further assess in vivo SPP1 expression, we developed *Spp1*-IRES-TdTomato (TdT) (*Spp1*^TdT^) reporter mice, which carry an IRES-TdT cassette in *Spp1* exon 7 retaining endogenous SPP1 expression (Extended Data Fig. 3a–c). Flow cytometric analysis of naïve animals revealed the expression of *Spp1-*TdT within 25.9% of pregated CD45^hi^ CD206^+^ PVM, in contrast to CD11b^+^ CD45^int^ CX3CR1^hi^ microglia that were almost devoid of TdT expression (0.9% of which were TdT^+^) (Fig. 2d–f). Further, only approximately 12% of total live TdT^+^ cells were CD140a^+^ (gene product of *Pdgfra*), in contrast to 20.5% for CD206^+^, highlighting PVMs as a predominant cellular source of SPP1 (Fig. 2e,f). Using high-resolution confocal imaging, we found that the distribution of SPP1-TdT was comparable to the SPP1 immunoreactivity detected by IHC (Fig. 1i), that is, along GLUT1^+^ vasculature of the hippocampus (Extended Data Fig. 3d). Further, we saw similar cellular localization of SPP1-TdT within CD206^+^ PVMs in the *Spp1*^TdT^ hippocampus (Fig. 2g). Finally, we used correlative light and electron microscopy (CLEM) to target and visualize the ultrastructure and environmental context of SPP1-TdT positive cells in the hippocampus (Fig. 2h, Extended Data Fig. 3e and Supplementary Video 1). CLEM identified SPP1-TdT expressing cells as lysosome-rich PVMs located within the basement membrane of the perivascular space (Fig. 2h and Extended Data Fig. 3e). CLEM and mRNA in situ localization identified PVMs as a source for SPP1 in mice, which translated to human tissue, where we found enrichment of SPP1 within the perivascular space of AD patients, occasionally overlaying with CD206^+^ cells (Fig. 2i). Collectively, our data suggest that SPP1 is predominantly expressed by CD206^+^*Cd163*^*+*^*Pf4*^*+*^ PVMs and *Pdgfra*^*+*^/CD140^+^ PVFs in the mouse hippocampus during onset of microglia–synapse engulfment. Similar to perivascular SPP1 upregulation in mice, we also found perivascular SPP1 expression in CA1 hippocampus of AD patient brains.

### SPP1 drives microglial engulfment of synapses in AD context

**Fig. 3:**
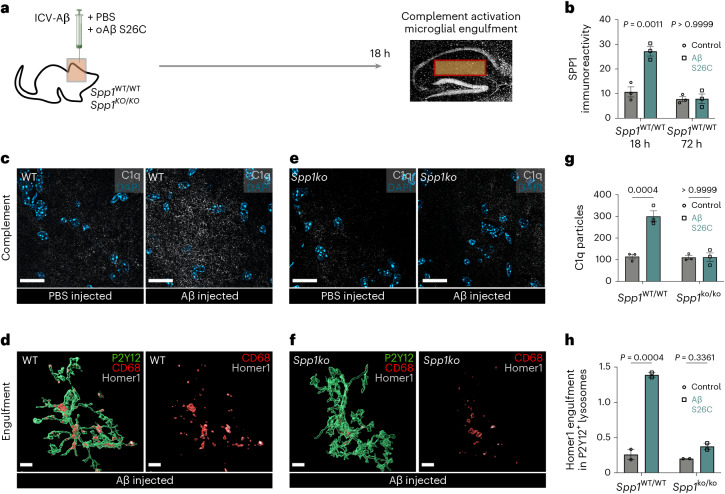
*Spp1* modulates complement activation and microglial synaptic engulfment upon acute oAβ challenge. **a**, Scheme illustrating ICV injection of S26C oAβ versus PBS in WT versus *Spp1*^*KO/KO*^ mice, 18 h before tissue collection and analysis. **b**, Quantification of SPP1 immunoreactivity within SLM of 3-month WT mice injected with oAβ versus PBS control, at either 18 h or 72 h post-ICV injection. One datapoint represents one ROI per mouse hippocampus, with total of *n* = 3 mice per genotype and time point, examined over one independent experiment. *P* values from two-way ANOVA, Bonferroni’s multiple comparison test. **c**, Representative images of C1q expression in SLM of 3-month PBS versus oAβ-injected WT mice. Scale bar represents 20 µm. **d**, Representative 3D reconstructed images showing Homer1 engulfment within CD68^+^ lysosomes of P2Y12^+^ microglia from WT mice injected with oAβ. Scale bar represents 5 µm. **e**, Representative images of C1q expression in *Spp1*^*KO/KO*^ mice injected with PBS versus oAβ. **f**, Representative 3D reconstructed images showing Homer1 engulfment within CD68^+^ lysosomes of microglia from *Spp1*^*KO/KO*^ mice injected with oAβ. Scale bar represents 5 µm. **g**, Quantification of C1q particles (puncta) in WT or *Spp1*^*KO/KO*^ mice treated with either PBS or oAβ, as in **c** and **e**. One datapoint represents average of one mouse, calculated from 3-4 ROIs per mouse from *n* = 3 mice, examined over 2 independent experiments. *P* Values from two-way ANOVA, Bonferroni’s multiple comparison test. **h**, Quantification of Homer1 engulfment in WT or *Spp1*^*KO/KO*^ P2Y12^+^ microglia, ICV treated with either PBS or oAβ, as in **d** and **f**. One datapoint represents average of 1 mouse, calculated from 5–11 individual P2Y12^+^ microglia per mouse from *n* = 2 animals examined over 2 independent experiments. *P* Values from two-way ANOVA, Bonferroni’s multiple comparison test. Data are shown as mean ± s.e.m. Source data

We next addressed the functional consequences of the SPP1 increase that spatiotemporally coincides with onset of microglia–synapse engulfment. To determine whether SPP1 has a role in microglial phagocytosis of synapses, we first performed intracerebroventricular (ICV) injections of oAβ in WT versus *Spp1*-deficient^20^ (*Spp1*^*KO/KO*^) mice. As we have previously shown, oAβ triggers microglia- and complement-mediated synapse engulfment^15^ (Fig. 3a). Concurrently, we observed a robust upregulation of SPP1 in the contralateral hippocampus of WT mice 18 h after ICV injection of oAβ (Fig. 3b). This upregulation coincided with microglial C1q activation and peak engulfment of Homer1-immunoreactive synapses by microglia in contralateral hippocampus of oAβ-injected WT mice (Fig. 3c,d,h). In contrast, oAβ failed to upregulate C1q or induce microglial synaptic engulfment in *Spp1*^*KO/KO*^ mice, suggesting that SPP1 is necessary for microglia–synapse engulfment.

**Fig. 4:**
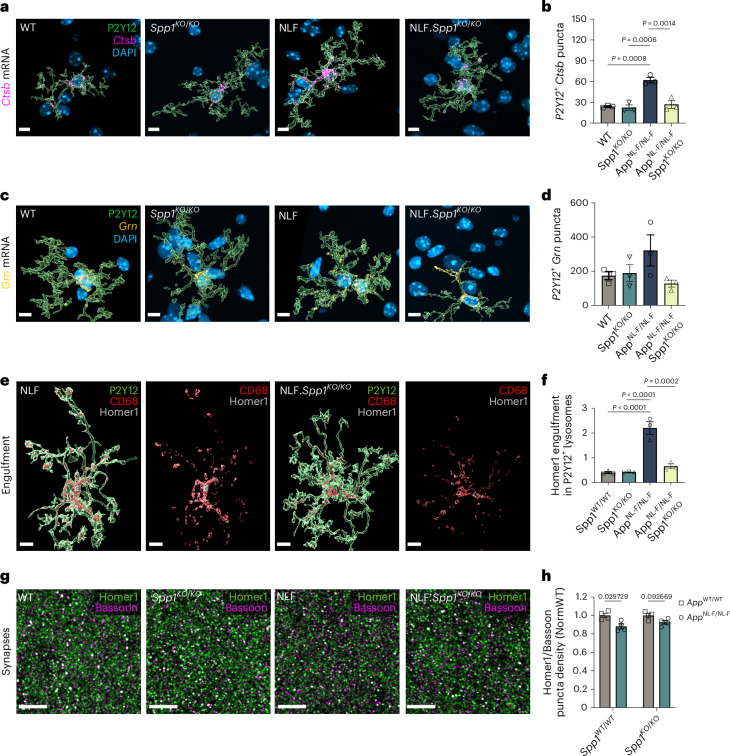
SPP1 drives microglial engulfment of synapses in AD context. **a**–**d**, Representative image of 3D reconstructed P2Y12^+^ microglia expressing phagocytic markers *Ctsb* (**a**,**b**) and *Grn* (**c**,**d**) assessed by smFISH-IHC in 6-month WT, *Spp1*^*KO/KO*^*, App*^NL-F^ and *App*^NL-F^·*Spp1*^*KO/KO*^ SLM. Scale bar represents 7 µm. Quantification of *Ctsb* (**b**) and *Grn* (**d**) mRNA levels expression within P2Y12^+^ microglia. One datapoint represents average of 1 mouse, calculated from 6 individual P2Y12^+^ microglia per mouse (**b**) and 3–6 microglia (**d**) from *n* = 3 mice per genotype examined over 2 independent experiments. *P* Values from two-way ANOVA, Bonferroni’s multiple comparison test for (**b**). **e**, Representative 3D reconstructed images showing Homer1 engulfment within CD68^+^ lysosomes of P2Y12^+^ microglia in 6-month *App*^NL-F^ versus *App*^NL-F^·*Spp1*^*KO/KO*^ SLM. Scale bar represents 7 µm. **f**, Quantification of Homer1 engulfment ratio in P2Y12^+^ microglia of WT versus *Spp1*^*KO/KO*^ versus *App*^NL-F^ versus *App*^NL-F^·*Spp1*^*KO/KO*^ mice. One datapoint represents average of 1 mouse, calculated from 7–9 individual P2Y12^+^ microglia per mouse from *n* = 3 mice examined over 2 independent experiments. *P* Values from two-way ANOVA, Bonferroni’s multiple comparison test. **g**, Representative super-resolution images of Homer1 and Bassoon puncta colocalization in 6-month WT, *Spp1*^*KO/KO*^, *App*^NL-F^ and *App*^NL-F^·*Spp1*^*KO/KO*^ SLM. Scale bar represents 5 µm. **h**, Quantification of Homer1/Bassoon colocalization density normalized to WT or *Spp1*^*KO/KO*^ accordingly. One datapoint represents the average of one animal (3–5 ROIs per animal) with a total of *n* = 4 animals per genotype. *P* < 0.029729 (WT) and 0.092669 (*Spp1*^*KO/KO*^) from multiple unpaired *t*-test, Bonferroni’s multiple comparison test. Data are shown as mean ± s.e.m. Source data

**Extended Data Fig. 4:**
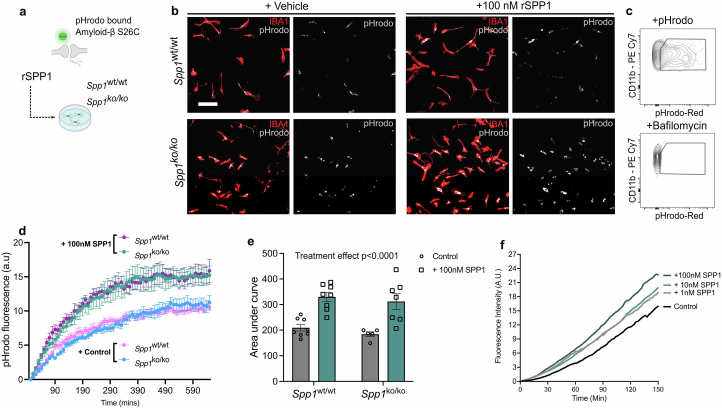
Primary microglia engulf synaptosomes through extrinsic SPP1. **(a)** Schematic illustrating *in vitro* engulfment assay whereby primary microglia isolated from WT or *Spp1*^*KO/KO*^ mice phagocytose S26C oAβ-treated synaptosomes that are tagged with pHrodo upon PBS or recombinant SPP1 treatment. **(b)** Representative images showing engulfment of pHrodo-synaptosomes by primary microglia isolated from WT mice (upper panel) or *Spp1*^*KO/KO*^ mice (lower panel), treated with vehicle (left) or 100 nM recombinant SPP1 (right). Data are representative of primary microglial cultures prepared from n = 6−8 neonates per genotype, examined over 2 independent experiments. Scale bar represents 100 µm. **(c)** Representative FACS plot showing pHrodo signal in CD11b^+^ cells collected from primary microglial culture (upper). No pHrodo signal was observed after Bafilomycin treatment, confirming lysosomal acidification as the source of pHrodo signal (lower). Data are representative of 2 independent experiments. **(d)** pHrodo fluorescence (arbitary fluorescence units) over 600 min (3–5 min intervals, synaptosomes added at t = 0) in primary microglia isolated from WT mice or *Spp1*^*KO/KO*^ mice, treated with control versus 100 nM recombinant SPP1. 1 datapoint represents 2–4 ROIs (approximately 40 microglia per ROI). Primary microglial cultures have been prepared from n = 5−8 neonates per genotype, examined over of 2 independent experiments. Data are shown as Mean ± SEM. **(e)** Area under curve quantification of pHrodo-synaptosome engulfment within primary WT versus *Spp1*^*KO/KO*^ microglia (d), treated with control versus 100 nM recombinant SPP1. 1 datapoint represents area under the curve (AUC) at 3 h of 2–4 ROIs (approximately 40 microglia per ROI). Primary microglial cultures have been prepared from n = 6−8 neonates per genotype, examined over of 2 independent experiments. *P* Values from two way ANOVA, Bonferroni’s multiple comparison test. Overall treatment effect F(1,24) = 43.29, *P* < 0.0001. Data are shown as Mean ± SEM. **(f)** Graph showing fluorescence intensity of pHrodo within primary WT microglial lysosomes over time (min), either in the presence of control or 1, 10 and 100 nM recombinant SPP1. Representative of 2 independent experiments. Source data

To test whether *Spp1* deficiency altered phagocytic signature in microglia in 6-month *App*^NL-F^ mice, we crossed the *Spp1*^*KO/KO*^ mice to *App*^NL-F^ mice (*App*^NL-F^·*Spp1*^*KO/KO*^). Using smFISH-IHC and 3D reconstruction, we observed an upregulation trend for Grn and a significant increase for *Ctsb*, encoding for progranulin and Cathepsin B, respectively, key components of the endolysosomal processing machinery in P2Y12^+^ microglia of *App*^NL-F^ animals (Fig. 4a–d)^14^. However, in the *App*^NL-F^·*Spp1*^*KO/KO*^ hippocampus, we found substantially decreased levels of *Grn and Ctsb* mRNA expression by P2Y12^+^ microglia, with Grn showing a downward trend (Fig. 4a–d). In fact, *Grn* and *Ctsb* expression levels were similar to WT and *Spp1*^*KO/KO*^ mice. These results suggest that microglia fail to adopt phagocytic states in *App*^NL-F^·*Spp1*^*KO/KO*^ mice as compared to those of *App*^NL-F^ mice, despite oAβ production. Further, we observed a sharp reduction of engulfed synapses inside P2Y12^+^ microglia of *App*^NL-F^·*Spp1*^*KO/KO*^ mice (Fig. 4e,f). These data altogether suggest that the absence of SPP1 prevents microglial phagocytosis as well as synaptic engulfment in response to amyloidosis (Figs. 3–4). We next tested whether microglia in *Spp1*^*KO/KO*^ mice display intrinsic defective phagocytic capacity. We assessed the ability of microglia to engulf synaptosomes in vitro using isolated primary microglia from postnatal WT versus *Spp1*^*KO/KO*^ mice (Extended Data Fig. 4a). We found that engulfment of oAβ-bound synaptosomes was similarly performed by microglia isolated from either genotype (Extended Data Fig. 4b–e). This suggests that diminished microglia–synapse engulfment in *Spp1*^*KO/KO*^ mice in vivo is not due to an inability of microglia to phagocytose or respond to oAβ, but likely results from defective extrinsic SPP1 signaling in the brain. Corroborating this hypothesis, the application of extracellular SPP1 was sufficient to promote microglia–synapse engulfment in *Spp1*^*KO/KO*^ primary microglia, demonstrating similar efficiency in engulfing synaptosomes compared to their WT counterparts upon SPP1 pretreatment (Extended Data Fig. 4d–f).

**Extended Data Fig. 5:**
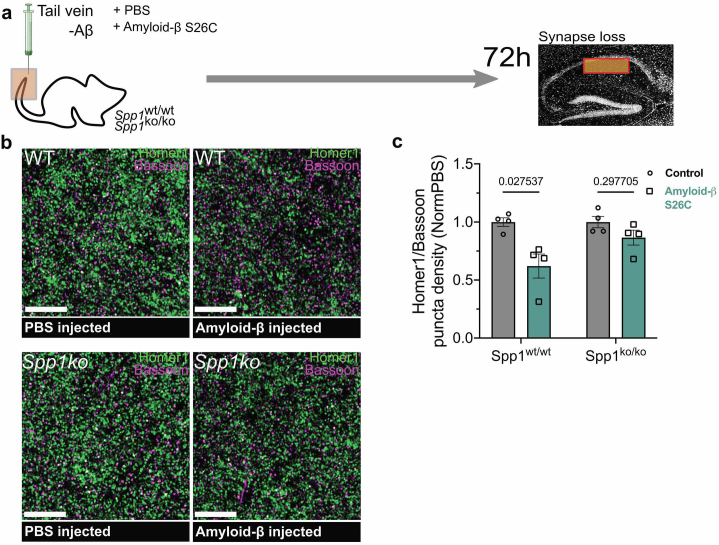
SPP1 deficiency prevents synapse loss upon oAβ challenge. **(a**) Scheme illustrating tail vein (TV) injection of 100ng S26C oAβ versus PBS in WT versus *Spp1*^*KO/KO*^ mice, 72 h before tissue collection and analysis. **(b)** Representative super-resolution images of Homer1 and Bassoon puncta colocalization in 3 mo WT and *Spp1*^*KO/KO*^ SR injected with oAβ versus PBS control, at 72 h post-TV injection. Scale bar represents 5 µm. (**c**) Quantification of Homer1/Bassoon colocalization density, represented as S26C oAβ-injected mice normalized to PBS injected mice. 1 datapoint represents the average of 1 animal (3–5 ROIs per animal, 40 cells per ROI) with a total of n = 4 mice per genotype. Data are from one experiment. *P* Values from multiple unpaired *t*-tests, Bonferroni’s multiple comparison test. Data are shown as Mean ± SEM. Source data

Finally, to determine the consequence of *Spp1* deficiency on synapse numbers in 6 mo *App*^NL-F^ animals, we used super-resolution microscopy to analyze presynaptic and postsynaptic markers (Bassoon and Homer1, respectively) in the hippocampal CA1 SLM of 6 mo *App*^NL-F^ mice (Fig. 4g,h). While we observed synaptic loss in colocalized Bassoon and Homer1-immunoreactive synaptic puncta density in *App*^NL-F^ versus WT mice, *Spp1* deficiency restored this synapse loss (Fig. 4h). Likewise, we observed that acute oAβ challenge failed to induce synapse loss in *Spp1*^*KO/KO*^ mice as it did in WT mice, suggesting that SPP1 is required for oAβ-induced synapse loss (Extended Data Fig. 5). Altogether, these results suggest that SPP1, likely secreted from PVMs in vivo in response to oAβ vasculature deposition, acts as an extrinsic signal to promote microglial engulfment of synapses and synapse loss in 6-month *App*^NL-F^ animals.

### *Spp1* regulates perivascular–microglial interaction networks

**Extended Data Fig. 6:**
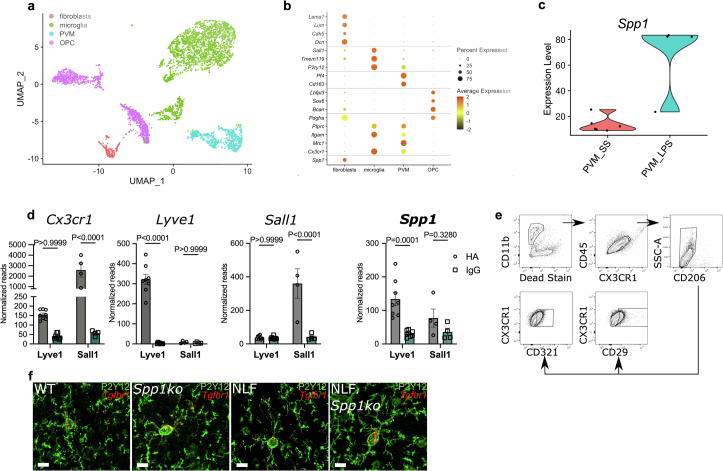
scRNA-seq and translatome analysis of perivascular cells and microglia. **(a)** UMAP plot of 4,238 cells, color-coded based on the inferred cell type. **(b)** DotPlot of gene expression for a series of known cell type markers for fibroblasts (*Lama1, Lum, Cdh5, Dcn*), microglia (*Sall1, Tmem119, P2ry12*), PVMs (*Pf4, Cd163*), and OPC (*Lhfpl3, Sox6, Bcan*). Expression of genes encoding for markers used for FACS of cells prior to scRNA-seq (*Pdgfra, Ptprc, Itgam, Mrc1, Cx3cr1*) and for *Spp1* is also included. Radius of dot is proportional to the percentage of cells expressing the gene, color is the scaled gene expression level. **(c)** Violin plot of *Spp1* expression reanalyzed from Jung-Seok Kim et al.^11^. **(d)** RiboTag-based translatome analysis of pooled oAβ and PBS-challenged brain homogenates of *Cx3cr1*^*ccre*^*:Lyve1*^*ncre*^ (n = 8 mice) and *Cx3cr1*^ccre^*Sall1*^ncre^:RiboTag-mice (n = 4 mice). Normalized reads of pan macrophage marker (*Cx3cr1*), PVM marker (*Lyve1*), microglia marker (*Sall1*) and *Spp1*. *P* Values from two-way ANOVA, Bonferroni’s multiple comparison test. Data are shown as Mean ± SEM. Data are from 1 independent experiment. **(e)** Gating strategy to identify CD321 *(F11r)* and CD29 (*Itgb1*) expression in microglia (CX3CR1^high^CD45^+^ CD206^−^ CD11b^+^). **(f)** Representative image of P2Y12^+^ microglia expressing *tgfbr1*, assessed by smFISH-IHC in 6 mo *App*^NL-F^ and *App*^NL-F^.*Spp1*^*KO/KO*^ SLM. Scale bar represents 10 µm. Data representative of n = 3 mice per genotype examined over 1 independent experiment. Source data

To obtain insight into how SPP1 may regulate interactions between perivascular cells, that is PVM and PVF, and parenchymal microglia in the *App*^NL-F^ hippocampus, we dissected hippocampi from 6-month WT, *Spp1*^*KO/KO*^, *App*^NL-F^, and *App*^NL-F^·*Spp1*^*KO/KO*^ animals and sorted CD45^high^CD11^int^CD206^+^ PVMs, CD140a^+^ PVFs and CD45^int^CD11^int^CX3CR1^high^ microglia for scRNA-seq analysis using the 10× Genomics platform. After quality control, we ran unsupervised clustering and annotated cell types based on expression of known marker genes (Extended Data Fig. 6a,b and Supplementary Table 2). Microglia expressed exclusive pan-markers such as *Sall1, Tmem119* and *P2ry12;* PVMs showed enrichment in *Mrc1*, *Pf4* and *Cd163* (Extended Data Fig. 6b)^11^. Of note, the sorted CD140a^+^ cells also included oligodendrocyte precursors (OPCs), which were discerned according to expression of OPC-specific markers including *Lhfpl3*, *Sox6* and *Bcan* (Extended Data Fig. 6b). Further, PVFs were positive for *Cdh5*, *Lama1* and *Dcn* that distinguished them from OPC. Interestingly, we did not observe *Spp1* mRNA expression levels in the sorted PVMs (Extended Data Fig. 2d). This conforms with previous scRNA-seq datasets from homogenized and isolated tissues^35^; however, translatome analysis of PVMs of brains of lipopolysaccharide (LPS) endotoxin challenged *Cx3cr1*^*ccre*^*:Lyve1*^*ncre*^:RiboTag mice have demonstrated the presence of *Spp1* in Lyve1^+^ PVMs (Extended Data Fig. 6c)^11,37^. Indeed, comparative RiboTag analysis of challenged split-Cre animals that target PVMs and microglia, respectively^11^, confirmed prominent *Spp1* expression in PVMs as compared to microglia (Extended Data Fig. 6d). The discrepancy of detected expression levels between scRNA-seq of isolated cells versus the RiboTag approach, which bypasses cell isolation and sorting^38,39^, and the highly sensitive smFISH methods suggest that *Spp1* signature within PVMs is highly regulated and closely associated to changes in its microenvironment, as earlier demonstrated for many other microglial transcripts^40^. It further suggests that *Spp1*^+^ PVMs likely do not survive isolation and highlights the relevance of studying SPP1 in an intact spatial context.

**Fig. 5:**
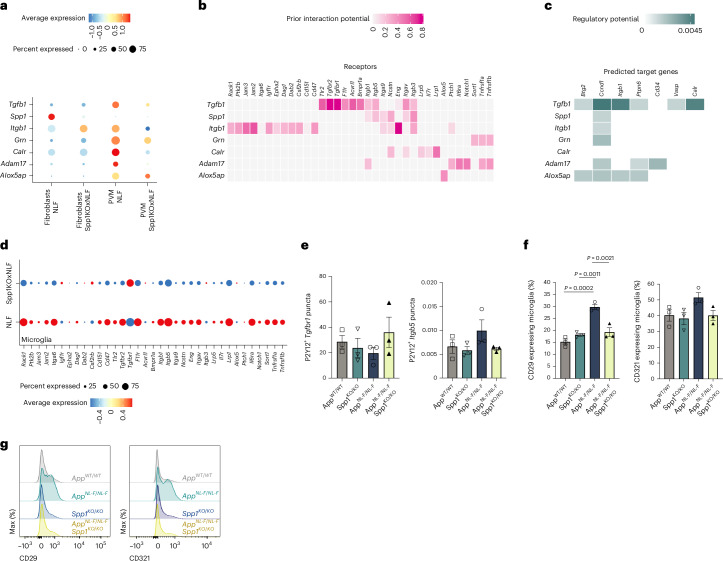
*Spp1* regulates perivascular–microglial interaction networks. **a**, Expression level of selected ligands expressed by cell types known to express *Spp1* (PVF and PVM), by cell type and genotype. Radius of dot is proportional to the percentage of cells expressing the gene; color is the scaled gene expression level. **b**, Predicted receptor genes for ligands represented in **a**, which show differential expression in microglia (receiver cells). Color represents the predicted interaction potential. **c**, Predicted target genes downstream of receptors identified in **b**, which show differential expression in microglia (receiver cells). Color represents the predicted regulatory potential. **d**, Expression of predicted receptor genes in microglia, by genotype. Radius of dot is proportional to the percentage of cells expressing the gene; color is the scaled gene expression level. **e**, Quantification of *Tgfbr1* and *Itgb5* mRNA levels expressed by P2Y12^+^ microglia assessed by smFISH-IHC in 6-month WT, *Spp1*^*KO/KO*^, *App*^NL-F^ and *App*^NL-F^·*Spp1*^*KO/KO*^ SLM. One datapoint represents average of 1 mouse, calculated from 3–7 individual P2Y12^+^ microglia per mouse from *n* = 3 animals examined over 1 independent experiment. *P* Values from two-way ANOVA. **f**, Quantification of NicheNet hits CD29 (*Itgb1)* and CD321 (*F11r)* on microglia (CX3CR1^high^CD45^+^CD11b^+^ CD206^−^) isolated from hippocampal homogenates of 6-month WT, *Spp1*^*KO/KO*^, *App*^NL-F^ and *App*^NL-F^·*Spp1*^*KO/KO*^ animals. One datapoint represents one individual mouse (microglia) pooled from *n* = 3 mice from one experiment. *P* values from two-way ANOVA, Bonferroni’s multiple comparisons test (CD29). **g**, Flow cytometry profiles of the protein expression intensity of CD29 and CD321 on microglia of the four genotypes. Data are shown as mean ± s.e.m. See also Extended Data Fig. 6 and Supplementary Table 2. Source data

Next, we used NicheNet on the scRNA-seq datasets to predict ligand–target links between interacting cells^28^. Specifically, we applied NicheNet to investigate how intercellular communication between PVM/PVF (ligand) and microglia (target) is altered by *Spp1* deficiency in 6-month *App*^NL-F^ mice (Fig. 5). We hypothesized SPP1 to function through paracrine and/or autocrine mechanisms, that is, modulating microglial function through direct or indirect signaling within the perivascular space. Among top predicted ligands affected in PVMs and PVFs by *Spp1* deficiency in 6-month *App*^NL-F^ hippocampus is transforming growth factor-beta 1 (TGF-β1), a cytokine critical for microglial development and previously shown to be modulated by SPP1 within fibroblasts^41,42^ (Fig. 5a–c). Other ligands include A Disintegrin and Metallopeptidase Domain 17 (ADAM17) and calreticulin, the latter being reported as an ‘eat-me’ signal driving macrophage phagocytosis of apoptotic cells^43^. Further, expression of *Itgb5, Itgb1 and Itgav*, encoding integrin receptors and subunits of the SPP1 receptor, were affected in microglia of *App*^NL-F^·*Spp1*^*KO/KO*^ versus *App*^NL-F^ mice, as expected^44^. Other top affected receptors in microglia were Tgfbr1, Tgfbr2 and protein tyrosine phosphatase Ptpn6 (Fig. 5b,c)^45,46^. We further validated these results via smFISH-IHC; as suggested by NicheNet, *Tgfbr1* and *Itgb5* expression levels showed a trend towards dysregulation in microglia of *App*^NL-F^·*Spp1*^*KO/KO*^ versus *App*^NL-F^ mice (Fig. 5e and Extended Data Fig. 6e,f). In addition, we confirmed decreased expression of CD29 (*Itgb1*) and a trending reduction in CD321 (*F11r*) in hippocampal microglia of *App*^NL-F^·*Spp1*^*KO/KO*^ compared to *App*^NL-F^ animals by flow cytometry (Fig. 5f,g and Extended Data Fig. 6f). Altogether, NicheNet revealed evidence for paracrine and autocrine crosstalk signals between the perivascular space and microglia in *App*^NL-F^ mice in the context of SPP1 signaling, with the majority of the top hits implicating potential alterations of microglial phagocytic states.

## Discussion

Microglia–synapse engulfment has been demonstrated to be relevant to synaptic loss and dysfunction in a wide variety of neurologic diseases across the lifespan, including neurodevelopmental and neuropsychiatric disorders, acute injury and virus-induced cognitive impairment, and multiple sclerosis and neurodegeneration^15,29,47–56^. Genetic studies in sporadic AD further highlight the importance of understanding microglial phagocytosis^14^. Therefore, our study demonstrating a functional role for SPP1 in microglia–synapse phagocytosis will likely have broad relevance to understanding neuroimmune mechanisms of synapse vulnerability in AD and other neurologic diseases involving synaptopathy.

Here we report a role for perivascular SPP1 as an upstream mediator of microglia–synapse engulfment in both genetic (*App*^NL-F^) and acute oAβ challenge mouse models. SPP1 is a multifunctional glycoprotein that was originally identified as a proinflammatory cytokine secreted by T cells and later found to be expressed in distinct tissue-resident macrophages linked with active clearance of apoptotic cells, chemotaxis and macrophage migration^18,57^. In the brain, SPP1 expression appears to be highly regulated in a spatiotemporal and cell type-specific manner, depending on context, age and brain region. In the perinatal and prenatal brain, *Spp1* is expressed by microglia and associates with axon tracts of the corpus callosum, in contrast to the adult brain where SPP1 expression is thought to be restricted to glutaminergic and GABAergic neurons in adult hindbrain^11,37,58–60^. Using various imaging tools including smFISH-IHC and CLEM in *Spp1*^TdT^ mice, we demonstrate that SPP1 expression in the hippocampus of healthy adult WT animals is restricted to the perivascular space, that is, *Pf4*, CD163^+^ and CD206^+^ PVM and to a much lesser extent, the closely neighboring PVFs. The finding that PVMs act as a major source of SPP1 upon oAβ challenge is consistent with the enrichment of ribosome-attached *Spp1* transcripts in Lyve1^+^ PVMs compared to Sall1^+^ microglia and is consistent with our earlier study that shows *Spp1* upregulation in Lyve1^+^ PVMs upon LPS challenge in adult mice^11^. Further, we found PVMs as the predominant cellular origin for SPP1 activation in early *App* models coinciding with Aβ aggregation along the vasculature. Of note, a recent study demonstrated early *Spp1* upregulation in spinal cord PVFs in a mouse model of amyotrophic lateral sclerosis, preceding microglial activation and onset of motor neuron loss^61^. This suggests that SPP1 activation might represent a conserved molecular response to perturbed perivascular homeostasis, potentially beyond Aβ pathology.

We further recapitulated the perivascular localization of SPP1 in postmortem human brains of AD patients. These results are in line with a recent single-nucleus transcriptomic study in vessel-associated structures of the human cortex that found *SPP1* expression in perivascular myeloid cells^62^. Our study thus raises an intriguing question of what could possibly trigger SPP1 elevation within the perivascular space. In AD patients, SPP1 levels are elevated in CSF^21–23^, which flows via the perivascular space toward the subarachnoid space. The cellular origin or the functional relevance of SPP1 increase, however, has been unclear. PVMs are distributed along the perivascular space and may have a critical role as sentinels in amyloidosis (our data here and ref. ^63^). Clearance of Aβ across the BBB represents an important homeostatic function, the impairment of which has been linked to exacerbated vascular as well as parenchymal deposition of Aβ^64,65^. Blood vessels therefore may represent a frequent, early and vulnerable site for Aβ deposition. In support of this, vascular pathology is commonly observed in AD, afflicting approximately 80% of patients^66^. Our findings of oAβ deposition along the vasculature in early stages of amyloidosis in *App*^NL-F^ mice further support this view. Indeed, it was postulated that early failure of perivascular drainage could contribute to parenchymal Aβ deposition and associated neuronal toxicity^67^. Furthermore, SPP1 has been linked to the clearance of vascular Aβ deposits in mice^68^. Altogether, these and our studies highlight Aβ deposition along the vasculature as a possible trigger for SPP1 upregulation in PVMs and PVFs. Future studies allowing cell-type specific fate mapping and mutagenesis of SPP1 in hippocampal PVM and PVF subsets will be needed to provide insight into the origin and fate of SPP1-expressing perivascular subsets.

Our observation that SPP1 signaling promotes synaptic engulfment by microglia in the hippocampus of AD models suggests that SPP1 functions to regulate phagocytic cell states. Indeed, in the absence of *Spp1*, microglia fail to upregulate key phagocytic and AD-relevant *Ctsb* in 6-month *App*^NL-F^ mice. Of note, phagocytosis induced by complement proteins including C3 has been considered protective against Aβ plaque load and neuronal loss in plaque-rich mouse models of AD. In line with our data, this suggests that the timing of phagocytic activity in different stages of disease might be critical to determine the protective versus detrimental nature of the outcome^15,69,70^. Another interesting question raised is how SPP1 modulates microglial phagocytosis in the presence of Aβ pathology. SPP1 functions as a secreted or intracellular isoform^71^. Based on our data, a direct effect of intracellular SPP1 on microglia function seems unlikely in our experimental setting. First, we found that microglia-intrinsic *Spp1* signaling is not required for the engulfment of Aβ-treated synaptosomes, where primary microglia derived from WT and *Spp1*^*KO/KO*^ mice exhibit similar engulfment capacities of synaptosomes in the presence of extracellular SPP1. This is consistent with work in primary gut macrophages from *Spp1*-deficient mice, whereby low doses of recombinant SPP1 restored phagocytic function toward opsonized bacteria, akin to what we observed for primary microglia and synaptosomes^72^. Second, we did not observe mRNA or protein SPP1 expression localized inside hippocampal microglia at the age of 6 months, yet the same cells fail to engulf synapses in *App*^NL-F^·*Spp1*^*KO/KO*^ mice or *Spp1*^*KO/KO*^ mice challenged with synaptotoxic Aβ. Third, we found increased protein levels of SPP1-immunoreactive puncta within the SLM parenchyma using super-resolution imaging, suggesting that the observed punctate signals are mostly extracellular. Altogether, these results suggest that SPP1 is secreted by PVMs and PVFs and influences microglia phagocytosis.

Precise mechanisms of how microglial engulfment is initiated by SPP1 are to be investigated. SPP1 could opsonize neuronal material for example damaged synapses for microglial phagocytosis^73^. It could also engage directly with the canonical receptors, α_v_β_3_, which are expressed on microglia^37^, to trigger downstream signaling pathways. Indeed, using NicheNet, we observed *Itgb3* and *Itgav*, which encode α_v_β_3_, to be downregulated in microglia of *App*^NL-F^·*Spp1*^*KO/KO*^ mice. Our NicheNet analysis further shows reduced expression levels of both *Tgfb1* in PVMs and its receptor *Tgfbr2* in microglia from *App*^NL-F^·*Spp1*^*KO/KO*^ mice, suggesting that SPP1 potentially coordinates perivascular–microglial crosstalk via autocrine TGF-β signaling. Indeed, SPP1 has been identified to promote autocrine TGF-β signaling in fibroblasts in a mouse model of muscular dystrophy, thereby promoting fibrosis by neighboring fibroblasts and macrophages^41^. Further, TGF-β is a major determinant of microglia maturation and homeostasis, and *Tgfbr2* deficient microglia demonstrate dysregulated expression of transcripts related to phagosome formation and immune activation^42,45^. In contrast to decreased *Tgfbr2*, we observed a trend towards increased *Tgfbr1* signaling in microglia of *App*^NL-F^·*Spp1*^*KO/KO*^ versus *App*^NL-F^ animals. It is tempting to speculate that this paradoxical down- and upregulation reflects compensatory mechanisms to counteract microglia phagocytosis induced by SPP1. Finally, TGF-β1 has been shown to be increased in CSF and perivascular space of AD patients, where its levels positively correlate with Aβ deposition along blood vessels, and TGF-β1-overexpressing mice show enhanced microglial phagocytosis and plaque amelioration in a complement-dependent manner^74,75^. Further studies are needed to gain better insight into the role of TGF-β signaling in governing autocrine and paracrine PVM-microglial crosstalk in AD. Other predicted *Spp1*-specific pathways in microglia of *App*^NL-F^ mice include *Calr*, encoding for calreticulin, a multifunctional chaperone protein that has been described as an ‘eat-me’ signal^43^. Altogether, our results suggest multiple mechanisms by which microglial phagocytosis could be modulated by perivascular SPP1 signaling. Determining molecular mechanisms of how these pathways may contribute to synaptic engulfment will be important to better understand multicellular events and crosstalk between perivascular space and the brain parenchyma.

Altogether, our study nominates perivascular SPP1 as a modulator for microglial phagocytosis, impacting microglia–synapse interactions and synaptic homeostasis. In peripheral tissues, insight into stroma–macrophage interactions demonstrates intricate and functionally relevant immune crosstalk that underlies critical tissue remodeling and homeostasis^76^. Likewise, our results highlight a potential role for perivascular–microglia interactions in the brain, and the presence of an SPP1-producing perivascular niche may offer opportunities to specifically manipulate and block microglia-mediated synaptic engulfment.

## Methods

### Mice

All experiments were performed in accordance with the UK Animal (Scientific Procedures) Act, 1986 and following local ethical advice. Experimental procedures were approved by the UK Home Office and ethical approval was granted through consultation with veterinary staff at University College London (UCL).

For all experiments, C57BL/6J (WT) mice were obtained from Charles River UK. *Spp1*^*KO/KO*^ (B6.129S6(Cg)-Spp1^tm1Blh^/J; stock 4936) and CX_3_CR-1^GFP^ (B6.129P2(Cg)-Cx3cr1^tm1Litt^/J; stock 5582) mice were obtained from The Jackson Laboratory. *App*^NL-F/NL-F^ mice were obtained from F. A. Edwards (Department of Neuroscience, Physiology & Pharmacology, UCL, UK)^30^. *Cx3cr1*^*ccre*^*:Sall1*^*ncre*^ (split-Cre) R26-LSL-tdTom:*Rpl22*^HA^ (RiboTag) and *Cx3cr1*^*ccre*^*:Lyve1*^*ncre*^R26-LSL-tdTom:*Rpl22*^HA^ homozygous animals were maintained in specific pathogen-free conditions and handled according to protocols approved by the Weizmann Institute Animal Care Committee as per international guidelines. All animals were housed under temperature-controlled (temperature, 23.1 °C; humidity, 30–60%) and pathogen-free conditions with 12 h light/12 h dark cycle with an ad libitum supply of food and water. Both male and female age-matched mice were used in this study: WT males and females, *Spp1*^*KO/KO*^ males, CX_3_CR-1^GFP^ males, *Spp1*^*tm1(tdTomato)Msasn*^ males, *App*^NL-F/NL-F^ males and *App*^NL-F^·*Spp1*^*KO/KO*^ males.

### *Spp1* reporter mouse model development

All animal work was approved by the Jackson Laboratory Animal Care and Use Committee and adhered to the standards of the Guide for the Care and Use of Laboratory Animals set forth by the NIH. The *Spp1*^*tm1(tdTomato)Msasn*^ mouse allele was generated using direct delivery of CRISPR–Cas9 reagents to mouse zygotes. An IRES-tdT construct was introduced in the mouse Spp1 gene (Ensembl Gene UID, ENSMUSG00000029304; Extended Data Fig. 3a). Analysis of genomic DNA sequence surrounding the target region, using the Benchling (www.benchling.com) guide RNA (gRNA) design tool, identified a gRNA sequence (AACAAGAAAAAGTGTTAGTG) with a suitable target endonuclease site at the stop codon of exon 7 of the mouse *Spp1* locus. *Streptococcus pyogenes* Cas9 (SpCas9) V3 protein and gRNA were purchased as part of the Alt-R CRISPR–Cas9 system using the crRNA:tracrRNA duplex format as the gRNA species (IDT). Alt-R CRISPR–Cas9 crRNAs (1072532, IDT) were synthesized using the gRNA sequences specified in the DESIGN section and hybridized with the Alt-R tracrRNA (1072534, IDT) as per the manufacturer’s instructions. A plasmid construct with a 2 kB 5′ homology arm ending 62 bp past the Spp1 stop codon in exon 7, IRES, tdT coding sequence, bGH poly(A) signal and 1.5 kB 3′ homology arm was synthesized by Genscript (Extended Data Fig. 3b). To prepare the gene editing reagent for electroporation, SpCas9:gRNA ribonucleoprotein complexes were formed by incubating Alt-R–SpCas9 V3 (1081059, IDT) and gRNA duplexes for 20 min at room temperature in embryo tested Tris–EDTA (TE) buffer (pH 7.5). Fertilized mouse embryos were generated via natural mating and cultured as described previously^77^. C57BL/6J (Stock 000664, The Jackson Laboratory) donor female mice (3–4 weeks of age) were superovulated by administration of 5 IU of pregnant mare serum gonadotrophin via intraperitoneal (ip) injection (HOR-272, ProSpec) followed 47 h later by 5 IU (ip) human chorionic gonadotrophin (hCG) (HOR-250, ProSpec). Immediately postadministration of hCG, the female was mated 1:1 with a C57BL/6J stud male and 22 h later checked for the presence of a copulation plug. Female mice displaying a copulation plug were sacrificed, the oviducts excised and embryos collected. Electroporation was performed as described in ref. ^77^. In brief, zygotes were treated with the acidic Tyrode’s solution (T1788, Millapore-Sigma) for 10 s and washed extensively in prewarmed M2 media (M7167, Millapore-Sigma). Zygotes were then placed in 10 μl drops of Opti-MEM media (3198570, Thermo Fisher Scientific–Gibco). Ten microliters of gene editing reagent solution, including the SpCas9/gRNA and the ssDO, was mixed with the Opti-MEM drops with the embryos and deposited into a 1 mm electroporation cuvette (45-0124, Harvard Apparatus). Electroporation was performed in an ECM830 Square Wave Electroporation System (45-0661, BTX). The electroporation setting was a 1 ms pulse duration and two pulses with 100 ms pulse interval at 30 V. Following the electroporation, a prewarmed 100 μl aliquot of M2 media was deposited into the cuvette with a sterile plastic pipette to recover the embryos. The zygotes were removed from the cuvette and washed in prewarmed M2 media. Embryos were immediately transferred into B6Qsi5F1 pseudopregnant female mice, an F1 hybrid strain produced by breeding C57BL/6J female mice with the inbred Quackenbush Swiss line 5 mouse strain^78^.

Founders were first assayed by short-range PCR with primer SR-FF 5′-TAATAATGGTGAGCAAGGGCGA-3′ and reverse primer SR-RR 5′-CTTTGATGACGGCCATGTTGTT-3′ within the reporter construct (see schematic). Positive founders were then screened by PCR: across the 5′ homology arm with primer 5′LR_F 5′-GAAAGTGCCTACTCGTGCCT-3′ and reverse primer 5′LR_R: CACATTGCCAAAAGACGGCA-3′; across the 3′ homology arm with primers 8431 GCATCGCATTGTCTGAGTAGGT and 3′LR_R: ccatcatggctttgcatgac; and for the presence of plasmid backbone with primers 8431: GCATCGCATTGTCTGAGTAGGT and 9581: AGCGCAACGCAATTAATGTG. Sanger sequencing was performed across the homology arm junctions and portions of the knocked-in reporter gene (Extended Data Fig. 3c).

Founders were selected that were positive by short-range PCR assays, had appropriate sequence across the homology arm junctions, were negative for the plasmid backbone and had correct sequence of the inserted construct. These were bred to C57BL/6J.

Once the line was established, mice were genotyped using a Taqman qPCR protocol run on a real-time PCR instrument (Roche LightCycler480). Forward primers for the wild-type allele (AAACACAGTTCCTTACTTTGCAT producing a 94 bp product) and the mutant allele (AGGATTGGGAAGACAATAGCA in the bGH poly A producing an 84 bp product) were combined with a common reverse primer (CACTGAACTGAGAAATGAGCAGT) using an annealing temperature of 60 °C. The wild-type probe (5′ HEX fluorophore label) used was TGTTAGTGAGGGTTAAGCAGGAATA and the knock-in probe (5′ FAM fluorophore label) used was ATGCGGTGGGCTCTATGG, each utilizing a black hole quencher on their 3′ end. These were run with an EndPoint protocol, imaging negative (quenched) fluorescent values at the completion of the cycling protocol.

This new line of *Spp1*-IRES-TdT mice is available as B6J.*Spp1*^*tm1(tdTomato)Msasn*^/J (stock 33731) from The Jackson Laboratory.

### ICV injections of S26C oAβ

S26C Aβ(1–40)_2_ dimers were purchased from Phoenix Pharmaceuticals^15^. Adult (2–3 months) mice were anesthetized with 4% isoflurane followed by maintenance at 1.5–2% isoflurane during surgery. Surgery was performed after head fixing in a stereotaxic frame (World Precision Instruments). Marcaine (0.025%) was applied locally, the skull was exposed by a single incision along the midline and a unilateral craniotomy was drilled with a 0.9-bit drill burr (Hager and Meisinger). Pulled long-shaft borosilicate pipettes (Drummond Scientific) were backfilled with mineral oil before loading with oAβ (1 ng µl^−1^) or sterile PBS vehicle. Four microliters total volume was injected into the right lateral ventricle (stereotaxic coordinates in millimeters from Paxinos and Franklin’s The Mouse Brain in Stereotaxic Coordinates, Fourth Edition; AP: −0.40, ML: 1.00, DV: −2.50) using a Nanofil 10 ml syringe (World Precision Instruments) connected to an UltraMicroPump-3 (World Precision Instruments) at a flow rate of 400 nl min^−1^. The pipette was left in place for 5 min after complete substance injection and slowly withdrawn to avoid backflow along the pipette track. The incision on the scalp was closed with Vetbond tissue adhesive (3 M). Subcutaneous carprofen (Carprieve, 5 mg g^−1^ body weight) and buprenorphine (Vetergesic, 0.1 mg g^−1^ body weight) diluted in 0.9% saline were administered perioperatively. Animals received carprofen (33.33 mg ml^−1^) in their drinking water until tissue collecting. The left hemisphere, contralateral to the injection site, was analyzed.

### Immunohistochemistry

Deeply anesthetized mice were transcardially perfused with 25–30 ml of filtered PBS, followed by 20 ml of 4% ice-cold ultrapure PFA (Generon, 18814-20). Brains were removed from the skull and fixed in 4% PFA (Generon) for 24 h at 4 °C. For cryoprotection, brains were rinsed in PBS for at least 2 h to remove excess PFA and placed in 30% sucrose for 48 h before embedding in optimal cutting compound. Fresh cryosections were blocked with 5% BSA, 0.2% Triton X-100 and 5% Donkey serum in PBS at room temperature for 90 min before incubation with primary antibodies at 4 °C overnight. All primary antibodies were diluted in a blocking buffer. After 4X PBS washes for 15 min each, sections were incubated with secondary antibodies in blocking buffer for 2 h at room temperature and then washed again with PBS. All secondary antibody aliquots were centrifugated at 15,000*g* for 15 min before being diluted in blocking buffer. The sections were then incubated for 5 min in DAPI diluted in PBS before being mounted either in Prolong Gold Antifade Mounting Medium (Thermo Fisher Scientific, P36930) for confocal imaging or in Prolong Glass Antifade Mountant (P36982) for STED imaging.

For immunostaining of C1q and synapses, 30 µm free-floating tissue sections were washed in PBS, followed by pretreatment in 1% Triton X-100 for 20. Sections were then blocked in 20% NGS, 1% BSA and 0.3% Triton, in PBS for 2 h followed by standard primary antibody incubation overnight at 4 °C. Sections are washed in 0.3% Triton X-100 in PBS for 30 min followed by secondary incubation for 4 h at room temperature and wash for 30 min before mounting.

### Postmortem human brain tissue

Brains were donated to the Queen Square Brain Bank (QSBB) for neurological disorders (UCL Queen Square Institute of Neurology). All tissue samples were donated with full, informed consent. Accompanying clinical and demographic data of all cases used in this study were stored electronically in compliance with the 1998 data protection act and are summarized in Supplementary Table 1. Ethical approval for the study was obtained from the NHS research ethics committee and in accordance with the human tissue authority’s code of practice and standards under license number 12,198, with an approved material transfer agreement. The cohort included pathologically diagnosed cases of AD (*n* = 6) and neurologically normal controls (*n* = 6). The level of AD pathology in all cases was assessed using current diagnostic consensus criteria^79,80^. The *APOE* genotype was also determined for each case as previously described^81^. Information regarding sex is included in Supplementary Table 1; however, sex was not taken into consideration when including patient samples. Findings did not apply to only one sex.

### Immunohistochemistry on postmortem human brain

Slides with 8 µm mounted tissue sections from the frontal cortex were incubated at 60 °C overnight. Sections were deparaffinized in xylene and rehydrated in decreasing grades of alcohol. Slides were incubated in methanol/hydrogen peroxide (0.3%) solution for 10 min to block endogenous peroxidase activity. For heat-induced antigen retrieval, slides were then transferred to a boiling solution of 0.1 M citrate buffer (pH 6.0) and pressure cooked at maximum pressure for 10 min. Nonspecific binding was blocked by incubating slides in 10% nonfat milk for 30 min at room temperature. Sections were incubated in anti-SPP1 antibody for 1 h at room temperature. After three gentle 5 min washes in tris-buffered saline with tween (TBS-T); slides were incubated for 45 min in biotinylated goat anti-mouse IgG secondary antibody (Vector Laboratories, BA-9200, 1:200). Slides were washed and incubated in avidin–biotin complex (ABC; Vector Laboratories) for signal amplification. The slides were then washed for a final time and 3,3′-diaminobenzidine was used as the chromogen and counterstained in Mayer’s hematoxylin (BDH). Finally, slides were dehydrated in increasing grades of alcohol (70%, 90% and 100% industrial methylated spirits), cleared in xylene and mounted.

Double immunofluorescence staining was carried out on the frontal cortex. Sections were prepared as detailed above up to the incubation of the anti-SPP1 antibody, secondary biotinylated goat anti-mouse and ABC. Antibody binding was visualized using a TSA Cyanine 3 amplification kit (PerkinElmer), which was applied to sections for 20 min at room temperature. After TBS-T washing, sections were incubated with anti-CD206 antibody for 1 h at room temperature and species-appropriate Alexa Fluor 658 secondary antibodies (Invitrogen, 1:1,000) for 2 h at room temperature to visualize the antibody. Sections were washed a final three times in TBS-T and mounted using Vectashield antifade mounting medium (Vector Laboratories).

### Image acquisition

Images were acquired using a Zeiss LSM800 confocal microscope (×40 objective, 1.3NA oil, ×20 objective 0.8-NA and 63×0.8-NA oil). Settings were kept constant for all sections in the same comparison group. Step size was determined using the optimal interval adjustment on the Zen blue software with a stack size of 10–14 µm for all on-slide IHC experiments and 6–10 µm for RNAScope experiments.

### Secreted SPP1 fluorescence intensity analysis

For quantification of secreted SPP1, Triton X-100 was excluded from the blocking buffer to retain signals of secreted and membrane-bound protein immune reactivity. To quantify SPP1 protein expression, images were processed in Fiji ImageJ (NIH)^82^. An automated ImageJ macro was created to analyze the signal intensity of each slice in the *z* stack and select the plane with the highest signal intensity. Background was subtracted with a rolling ball radius of 10 pixels. Images were thresholded with consistent thresholding parameters and made into binary images based on intensity. As pixel intensity information has been translated into area, the particle analysis function was used to quantify the total immune-reactive area.

### Antibodies

For immunostaining, all the antibodies used were rabbit anti-mouse C1q (Abcam, ab182451; Clone 4.8, 1/200), goat anti-mouse SPP1 (Bio-Techne, AF808; 1/50), rabbit anti-mouse IBA1 (Wako Chemicals, 019-19741; 1/500), rabbit anti-mouse GLUT1 (Merck Millipore, CBL242; 1/10,000), rat anti-mouse CD68 (Bio-Rad, MCA1957; Clone FA-11, 1/200), rabbit anti-mouse P2Y12 (Anaspec, AS-55043A; 1/500), rat anti-mouse CD206 (Bio-Rad, MCA2235; Clone MR5D3, 1/400), NAB61 (kindly provided by Virginia M-Y Lee, 1/500), HJ5.1 (kindly provided by John R. Cirrito, 1/500), chicken anti-mouse Homer1 (Synaptic System, 160 006; 1/500), mouse anti-mouse 6E10 (Biolegend, 803001; Clone 6E10, 1/200), mouse anti-mouse 4G8 (Biolegend, 800702; Clone 4G8, 1/200), rat anti-mouse CD140a (Thermo Fisher Scientific, 14-1401-82; Clone APA5, 1/50), rabbit anti-mouse Bassoon (Synaptic System, 141 003; 1/200) and Lyve1 (Abcam, ab14917; 1/500). Secondary antibodies used were a combination of Alexa Fluor 488, 594 and 647 (1/400, Jackson ImmunoResearch and Thermo Fisher Scientific) chosen from goat anti-rabbit, goat anti-rat, goat anti-mouse, donkey anti-goat, donkey anti-rat, donkey anti-mouse and donkey anti-rabbit secondaries. For flow cytometry, BUV395 CD45 (BD Biosciences, 564279; 1/400), Pe-Cy7 CD11b (BD Biosciences, 552850; 1/400), BV421 CX3CR1 (Biolegend, 149023; 1/400), PE CD140a (Miltenyi, 130-102-502, 1/50), APC CD140a (Miltenyi, 130-102-473, 1/50), APC CD206 (Biolegend, 141708, 1/400), FITC CD29 (Biolegend; 102205, 1/200), FITC CD206 (Biolegend; 141704, 1/400) and BV711 CD321 (BD Biosciences, 745405; 1/200) were used.

### In vivo microglial engulfment analysis

Engulfment analysis was performed as previously described^15^. Thirty micrometers of free-floating tissue sections were immunostained with IBA1, CD68 and Homer1. For each mouse, four to six regions of interest within CA1 SLM were acquired using a 63 × 1.4-NA objective on a Zeiss 800 microscope. Next, 60–80 *z*-stack planes were taken with 0.27-μm spacing and raw images were processed in Imaris (Bitplane) for analysis, after background subtraction of Homer1 channel. P2Y12^+^ cells and CD68 lysosomes were surface rendered with 0.25-μm and 0.1-μm smoothing, respectively. A mask was applied within P2Y12^+^ CD68^+^ reconstructed lysosomes for Homer1, and the percentage engulfment of Homer1 within lysosomes was calculated using the following formula: volume of engulfed material (Homer1 within CD68)/total microglial volume × 100.

### Super-resolution imaging and synapse analysis

Super-resolution synapse images were acquired on a Zeiss LSM 880 microscope with Airyscan detector using a 63x, 1.4-NA oil immersion Plan-Apochromat objective (theoretical maximum resolution—140 nm lateral, 350 nm axial). A zoom factor of 1.8× and a 37 × 37 × 144 nm voxel pixel size. The Airyscan detector was aligned before imaging each new slide. Three regions of interest of 1.15 µm were acquired in the center of the hippocampal CA1 stratum lacunosum moleculare for each brain section. Super-resolution synapse images were processed in Zen Black using 3D Airyscan Processing. Imaris software was used for presynaptic and postsynaptic puncta detection, using intensity centers to maximize the detection of immunoreactive spots. Presynaptic and postsynaptic spots were colocalized using a MATLAB colocalization script (Colocalize Spots XTension), using a colocalization distance of 0.25 μm between spot centers. Synapse density is shown as *App*^NL-F^ mice normalized to WT mice, and *App*^NL-F^·*Spp1*^*KO/KO*^ normalized to *Spp1*^*KO/KO*^ mice.

### smFISH (RNAscope) and smFISH combined with IHC (smFISH-IHC)

To detect single RNA molecules, the RNA probes for *Spp1* (435191), *C1q* (441221), *Bin1* (529541), *Ctsb* (561541), *Grn* (422861), *Cd163* (406631), *Pdgfra* (480661), *Pf4* (502391), *Tmem119* (472901), *Tgfbr1* (406201) and *Itgb5* (404311) were purchased from advanced cell diagnostics. RNAscope was performed following the manufacturer’s protocol in RNAscopeFluorescent Multiplex Assay (320293). 15 μm frozen brain sections were collected on Superfrost Plus GOLD Slides (Thermo Fisher Scientific, K5800AMNZ72) and dried overnight at 40 °C. Briefly, slides were incubated in H_2_O_2_ for 4 min at room temperature and washed in RNAse-free water. Slides were placed in boiling target antigen retrieval for 4 min, dehydrated in 100% ethanol for 5 min and treated with Protease Plus for 15 min at room temperature before probe incubation. Further protocol as described by the manufacturer, and post-RNAscope immunostaining as standard immunohistochemistry procedure.

### RNA isolation, reverse transcription and RT-qPCR

Mice were anesthetized and perfused with ice-cold PBS. Next, fresh cortex and hippocampus were dissected and homogenized with TissueLyser II (QIAGEN) using the mRNA easy mini kit (QIAGEN) as described by the manufacturer. Next, RNA purity and concentration were assessed by Nanodrop. mRNA was converted to cDNA using the qScript cDNA SuperMix reverse transcription kit as described by the manufacturer (95048, Quantabio). For RT-qPCR, 12 ng of cDNA was loaded in triplicates per gene in a total volume of 20 μl using the SYBR green PCR master mix as described by the manufacturer (4309155, Thermo Fisher Scientific). The reaction was run using a LightCycler 96 Instrument (Roche) with white 96-well plates (04729692001, Roche). Triplicate Ct values were averaged and data are shown as respective to the geomean of three housekeeping genes (*Actb, Gapdh* and *Rpl32*) using the Ct delta method (2–∆∆Ct). Primers purchased from IDT were used at a concentration of 200 nM. *Actb*, forward—CATTGCTGACAGGATGCAGAAGG, reverse—TGCTGGAAGGTGGACAGTGAGG; Gapdh, forward—CATCACTGCCACCCAGAAGACTG, reverse—ATGCCAGTGAGCTTCCCGTTCAG; *Rpl32*, forward—ATCAGGCACCAGTCAGACCGAT, reverse—GTTGCTCCCATAACCGATGTTGG. *Spp1*, forward—ATC TCACCATTCGGATGAGTCT, reverse—TGTAGGGACGATTGGAGTGAAA

### Microglia isolation for flow cytometry and FACS sort

PBS-perfused brains were quickly isolated from the skull and the hippocampus was dissected on ice using chilled instruments. For scRNA sequencing, brains were perfused with inhibitor cocktail including Actinomycin D (5 μg ml^−1^) and triptolide (10 μM). Next, single-cell suspension was prepared using the Adult Brain Dissociation kit from Miltenyi Biotec (Bergisch Gladbach, Germany), according manufacturer’s instructions and ref. ^25^. Afterwards, cell suspension was filtered through a 70 μM cell strainer before mixing with debris removal solution. Cells were centrifuged (300*g* for 10 min), washed with ice-cold FACS buffer (PBS, 2% FBS, 0.78 mM EDTA) and incubated for 30 min at 4 °C with FACS buffer containing Fc block (BD Biosciences) and primary antibody mix. Flow cytometry data were analyzed using FACSDiva software 4.0 and FlowJo 10 software (Treestar).

### Primary microglia isolation and culturing

For generation of microglial primary cultures, P0 WT or *Spp1*^*KO/KO*^ mice were decapitated, the brain was dissected from the skull and meninges were removed in ice-cold HBSS with 5% FBS. Eight to ten mice were pooled per culture preparation. Tissue was homogenized first with 2 ml pipettes (15 strokes) in 15 ml falcon tube and subsequently transferred to a prewet 50 ml tube with 70 μM strainer. The 15 ml tube was washed with HBSS and then put through filter to ensure all tissues were collected. The supernatant was removed and the cell pellet was resuspended in ice-cold 35% isotonic percoll. The interface was carefully created with HBSS. The samples were centrifuged for 40 min at 4 °C at 2,800*g* with no break and with slow acceleration and deceleration. The myelin layer and supernatant layers were aspirated, and the cell pellet was washed in HBSS. Cells were centrifuged and resuspended in 1 ml microglial media (DMEM F12 (Gibco), 5% FBS (Gibco), 1% pen-strep (Gibco), 50 ng ml^−1^ CSF1 416-ML-010/CF (RnD Systems), 50 ng ml^−1^ TGFb1 7666-MB-005/CF (RnD Systems) and 100 ng ml^−1^ CX3CL1 472-FF-025/CF (RnD Systems)) for cell counting. Cells were used within 7–10 d of plating.

### Isolation of synaptosomes and conjugation to pHrodo

Three to five 8-week-old animals were pooled per experiment. In brief, mice were intracardiac perfused with 10 ml ice-cold PBS. The hippocampi and cortices were dissected on ice. Tissue was weighed and homogenized in five volumes of sucrose homogenization buffer (5 mM HEPES pH 7.4, 320 mM sucrose and 1 mM EDTA) using a Dounce homogenizer with 15–20 strokes. The homogenate was centrifuged at 3,000*g* for 10 min at 4 °C and the supernatant was saved as total homogenate fraction (THF). The THF was centrifuged again at 14,000*g* for 12 min at 4 °C and supernatant was saved as cytosolic fraction. The pellet was carefully resuspended in 550 μl of Krebs–Ringer buffer (KRB: 10 mM HEPES, pH 7.4, 140 mM NaCl, 5 mM KCl, 5 mM glucose and 1 mM EDTA) and 450 µl of Percoll solution (for a final concentration of 45%). The solution was mixed by gently inverting the tube and an interface was slowly created with 400 µl of KRB. After centrifugation at 14,000*g* for 2 min at 4 °C, the synaptosomal fraction was recovered at the surface of the flotation gradient and carefully resuspended in 1 ml of KRB to wash. The functional synaptosomal preparation was centrifuged at 14,000*g* for 1 min at 4 °C. Fresh mouse synaptosomes were immediately divided into Eppendorfs at 2–2.5 mg of protein and resuspended in total 1 ml KRB in 50 nM of oAβ 40-S26C dimer or PBS as control and left overnight at 4 °C on nutator. Synaptosomes were then centrifuged at 14,000*g* for 1 min at 4 °C, supernatant was discarded and synaptosomes were washed in 1 ml PBS, after which they were centrifuged at 14,000*g* for 1 min at 4 °C to obtain oAβ-synaptosomes and control-synaptosomes. Briefly, 1 mg of synaptosomes were left at room temperature on nutator for 2 h in sodium bicarbonate 0.1 M with pHrodo Red, succinimidyl ester at a concentration of 1 mg ml^−1^. After conjugation, synaptosomes were centrifuged at 14,000*g* for 1 min and resuspended for use in the in vitro synaptosome engulfment assay.

### In vitro synaptosome engulfment assay

Primary mouse microglia were treated with 1 µg of pHrodo-conjugated S26C oAβ-treated synaptosomes. Plates were then placed in a CD7 with the incubator at 37 °C and 5% CO_2_. Fluorescent (594 nm and 647 nm) and brightfield (oblique and phase) images were acquired at a ×20 objective (×0.5) at intervals of 3–5 min. A three-slice *z* stack was taken at a 1.5-μm interval to ensure that imaging was within focus throughout the imaging session; however, one plane was used for analysis. For analysis, the *z*-profile axis was plotted for respective pHrodos on ImageJ with respect to time. Fluorescence intensity at *t* = 0 was subtracted from subsequent time frames.

### 3D-τ-STED/STED-FLIM

For visualization of secreted SPP1 in mouse and human tissue, tissue sections were imaged with Leica STELLARIS 8 STED microscope using the ×100 objective (1.4 NA oil) (Leica Microsystems). Tissue sections were imaged at least 24 h after being coverslipped with mounting medium to avoid discrepancies in fluorescence lifetime within each section. STED microscope with Fluorescence Lifetime Imaging (STED-FLIM) was used to visualize secreted SPP1 in the extracellular space and fluorescence lifetime information was used to gate fluorescence signals. Alignment between STED laser and excitation laser was performed before each imaging session. A 775 nm STED laser was used to generate a doughnut beam to silence the peripheral fluorophores from Alexa Flour 647 photoexcitation to achieve subdiffractional resolution. For all STED images, pixel size was limited to at most 50 nm. STED laser intensity was set at 20%. All images were taken with a step size of 0.15 μm. Fluorescence signal was time-gated from −0.5 ms to 4.5 ms. For analysis, raw images were processed by thresholding, followed by binary image transformation to match ROI with intensity. Finally, fluorescence intensity was measured by the particle analysis function in ImageJ (NIH).

### Volume correlative light and electron microscopy with array tomography

Mice were perfusion fixed with 4% PFA (EM grade) in ice-cold PBS as described above, the brain was dissected and left in 4% PFA in PBS overnight at 4 °C. The following day, coronal vibratome slices (100 μm) of the brain were collected and slices containing clear cross sections of the hippocampus were manually trimmed to minimally contain the hippocampus and ensure that the tissue piece was asymmetric. Low-resolution maps of the entire hippocampus tissue piece were taken using a confocal Zeiss LSM800 and ×10 lens with montaging for gross mapping and identification of regions of interest. High-resolution (63 × 1.4 NA) confocal stacks were taken of regions of interest. Slices were fixed further with 2% formaldehyde/1.5% glutaraldehyde in 0.1 M sodium cacodylate, before being processed for volume EM using a modified protocol based on the NCMIR protocol^83^. Briefly, tissue slices were incubated in 1% osmium tetroxide/1.5% potassium ferricyanide for 1 h at 4 °C, before being washed and left in 0.1 M sodium cacodylate in a fridge overnight. The following day, tissue was incubated in 1% thiocarbohydrazide for 15 min at 60 °C, 2% osmium tetroxide for 30 min, 1% uranyl acetate for 30 min and Walton’s lead aspartate for 30 min at 60 °C, with numerous distilled water washes between each step. The samples were consequently dehydrated through an ethanol series, embedded in Epon resin and baked overnight at 60 °C. Using the maps acquired by light microscopy, the block was trimmed down to the approximate region of interest and serial sections were collected on ITO-coated coverslips using an ultramicrotome (Leica) and diamond knife (Diatome). Coverslips were mounted on SEM stubs using carbon stickies and silver DAG and array tomography serial back scattered electron images (5 nm pixels) were acquired for each CLEM cell (*n* = 4) using Atlas 5 software and a Gemini 300 SEM (Zeiss) operating in high vacuum, at 4.5 kV with tandem decel operating at 3 kV. Serial images were registered using TrakEM2 (ref. ^84^) in Fiji (NIH), aligned with the confocal images in Photoshop (Adobe) using nuclei as unbiased fiducials and 2/3D reconstructed using Amira (Thermo Fisher Scientific).

### scRNA-seq library preparation, expression and NicheNet analysis

For scRNA-seq, we pooled tissue from four mice for each genotype, to be able to sort rare cell populations. Indeed, from one such preparation, we could isolate 5,000 microglia but only limited PVMs (200) and PVFs (350) on average per genotype. For each cell type and each genotype, we prepared a single scRNA-seq library, and we validated the findings using orthogonal methods such as smFISH and flow cytometry.

Single-cell libraries were prepared using 10X Genomics Chromium Next GEM Single Cell 3′ kit v3.1, according to the manufacturer’s instructions. Cells were loaded on the chromium chip according to Supplementary Table 2; because sorted PVMs and fibroblasts were in low abundance, we loaded the whole sample, while for microglia cells we aimed for 5,000 cells per sample. Libraries were sequenced using an SP flowcell on an Illumina NovaSeq S6000 instrument, with sequencing parameters recommended by 10X Genomics, aiming for 40,000 reads per cell. Raw FASTQ files were preprocessed using 10X Genomics CellRanger (version 6.0) and the mm10 mouse reference genome. Filtered gene expression matrices obtained from CellRanger were imported in R (version 4.1.2) and analyzed using the Seurat (version 4.0.6) and NicheNet (version 1.0.0) packages^28^. Cells with more than 10% of reads aligning to mitochondrial transcripts were deemed to be low quality or damaged and removed from the dataset. Cells remaining after each filtering step are reported in Supplementary Table 2. Identification of high variable genes, PCA and clustering were performed using Seurat functions. Cluster annotation was performed considering the expression of a set of known cell type markers (Extended Data Fig. 6). Differential expression analysis between pairs of conditions was performed with Seurat’s FindMarkers function using the Wilcoxon Rank Sum test. Comparisons between selected cell populations were performed using NicheNet functions and standard workflow.

### Ribosome immunoprecipitation (IP)

Brains were extracted from mice and homogenized in ice-cold homogenization buffer (50 mM Tris, pH 7.4, 100 mM KCl, 12 mM MgCl_2_, 1% NP-40, 1 mM DTT, 1:100 protease inhibitor (Sigma-Aldrich), 200 units per ml RNasin (Promega) and 0.1 mg ml^−1^ cycloheximide (Sigma-Aldrich) in RNase-free DDW) 10% wt/vol with a Dounce homogenizer (Sigma-Aldrich) until the suspension was homogeneous. Cell debris was removed by transferring 1 ml of homogenate and centrifuging at 10,000*g* and 4 °C for 10 min. Next, 10 μl of supernatant was removed for ‘input’ analysis and 10 μg of anti-HA.7 antibody (H9658, Sigma-Aldrich) or 10 µg of mouse monoclonal IgG1 antibody (Merck, PP100) was added to the supernatant, followed by 4 h of incubation with slow rotation in a cold room at 4 °C. Afterward, Dynabeads Protein G (Thermo Fisher Scientific) were equilibrated to homogenization buffer by washing three times. Samples were then incubated for 4 h with antibody and afterward beads were added for overnight incubation at 4 °C. Afterward, samples were washed three times with high-salt buffer (50 mM Tris, pH 7.4, 300 mM KCl, 12 mM MgCl_2_, 1% NP-40, 1 mM DTT, 1:200 protease inhibitor, 100 units per ml RNasin and 0.1 mg ml^−1^ cycloheximide in RNase-free DDW) for 5 min. At the end of the washes, beads were magnetized and excess buffer was removed, 100 µl Lysis Buffer was added to the beads and RNA was extracted with Dynabeads mRNA Direct purification kit (Thermo Fisher Scientific). RNA was eluted in 6 μl H_2_O and taken for RNA sequencing.

### Bulk RNA sequencing for ribosome IP samples

mRNA was captured with Dynabeads oligo(dT) (Life Technologies) according to the manufacturer’s guidelines. Qubit fluorometer (Life Technologies) technology was used to calculate library concentration and mean molecule size was determined with a 2200 TapeStation instrument. Next, libraries were sequenced using Illumina NextSeq-500. Raw reads were mapped to the genome (NCBI37/mm10) using hisat (version 0.1.6). Only reads with unique mapping were considered for further analysis. Gene expression levels of genes of interest (*Lyve1, Cx3cr1, Sall1* and *Spp1*) were calculated using the User-friendly Transcriptome Analysis Pipeline^85^. Next, DESeq2 R-package was used for normalization and differential expression analysis and pValue was adjusted by multiple gene testing <0.05.

### Statistics

All statistical analysis was performed in Prism (GraphPad Software, Version 9.3.1 or R (v.4.3.2)) and a complete overview is provided in Supplemental Table 3. Outliers were identified and removed from the dataset using Graphpad Prism (ROUT, Q = 1%). Normal distribution and equality of variance of the residuals were tested using the Shapiro-Wilk normality test and the *F*-test, Spearman’s test or Bartlett’s test, using significance level α = 0.05. To stabilize variances on heteroscedastic residuals, we performed log_10_ transformations on the data for Fig. 4d, 4f and Extended Data Fig. 4e before fitting the two-way ANOVA model (linear data was used for plotting to facilitate interpretation of the outcome). Two groups were compared using two-tailed unpaired Student’s *t*-test, two-tailed Mann-Whitney U tests or Welch’s *t*-test, depending on the data structure. To compare more than two groups (PVMs, microglia and PVFs), one-way ANOVA with Bonferroni’s multiple comparison post hoc test was used. Two-way ANOVA with Bonferroni’s multiple comparison test was used to assess the impact of Aβ exposure (*App*^NL-F^ genotype or oAβ injection) and Spp1 genotype (*Spp1*^WT/WT^, *Spp1*^KO/KO^), and the impact of recombinant SPP1 on both *Spp1*^WT/WT^ and *Spp1*^KO/KO^ mice. Due to the technical approach in synapse analysis studies, the analyses were performed in pairs, *App*^NL-F/NL-F^ mice were normalized to WT mice and *App*^NL-F/NL-F^·*Spp1*^KO/KO^ mice were normalized to *Spp1*^KO/KO^ mice, or oAβ injected mice were paired with control injected mice. As such, we performed two separate two-tailed unpaired *t*-tests between the sets, to which a Bonferroni correction for multiple comparisons was applied to reduce the likelihood of a type I error. For brain region (hippocampus vs. cerebellum) vs. genotype (WT vs. *App*^NL-F^) analysis, we used a linear mixed-effect model with multivariate *t*-distribution post-hoc, using mouse as a nested variable to account for inter-mouse variability [R packages lme4 (v.1.1-35.2), emmeans (v.1.10.0), lmerTest (v. 3.1-3)]. All data is presented as mean +/– SEM, statistical significance was set at α = 0.05. Data in graphs are presented as mean ± s.e.m. Statistical methods were not used to predetermine study sizes but were based on similar experiments previously published. Experiments were blinded to the genotype of the animal as well as the treatment of the animal. Experiments involving human sections were blinded to the demographics of the patients. Analysis of flow cytometry data of *Spp1*^TdT^ mice was performed in an unblinded fashion, for compensation purposes.

### Reporting summary

Further information on research design is available in the Nature Portfolio Reporting Summary linked to this article.

## Online content

Any methods, additional references, Nature Portfolio reporting summaries, source data, extended data, supplementary information, acknowledgements, peer review information; details of author contributions and competing interests; and statements of data and code availability are available at 10.1038/s41593-023-01257-z.

## Supplementary information

Reporting Summary

Supplementary Tables Supplementary Table 1—Case demographics for control and AD cases used in this study. Supplementary Table 2—Cell numbers of isolated and sequenced PVM, PVF and microglia of 6 mo wild-type, *App*^NL-F^, *Spp1*^*KO/KO*^ and *App*^NL-F^·*Spp1*^*KO/KO*^ mice after CellRanger filtering and QC filtering.

Supplementary Tables Supplementary Table 3—Overview of all the statistical tests used per figure in this study.

Supplementary Video Supplementary Video 1—Video showing back scattered electron images of serial sections of SPP1-TdT PVM shown in Fig. 2h. The manually segmented nuclei of the SPP1-TdT positive cell is shown highlighted in blue, to mark the SPP1-TdT correlated cell. Video width is 60 µm.

## Source data

Source Data Fig. 1 Raw data.

Source Data Fig. 2 Raw data.

Source Data Fig. 3 Raw data.

Source Data Fig. 4 Raw data.

Source Data Fig. 5 Raw data.

Source Data Extended Data Fig. 1 Raw data.

Source Data Extended Data Fig. 4 Raw data.

Source Data Extended Data Fig. 5 Raw data.

Source Data Extended Data Fig. 6 Raw data.

## Data Availability

The scRNA-seq data generated in this study are deposited at ArrayExpress (BioStudies, Annotare 2.0) under accession number E-MTAB-11918. The RiboTag-based RNA-seq data generated in this study are deposited at ArrayExpress (BioStudies, Annotare 2.0) under accession number E-MTAB-12515. Source data are provided within this paper. Raw data used in this study and requests for resources and reagents are available from the corresponding author upon request. Source data are provided with this paper.
