# Author Correction: Perivascular cells induce microglial phagocytic states and synaptic engulfment via SPP1 in mouse models of Alzheimer’s disease

**DOI:** 10.1038/s41593-025-02197-6

**Published:** 2026-02-17

**Authors:** Sebastiaan De Schepper, Judy Z. Ge, Annerieke Sierksma, Gerard Crowley, Laís S. S. Ferreira, Dylan Garceau, Christina E. Toomey, Dimitra Sokolova, Javier Rueda-Carrasco, Sun-Hye Shin, Jung-Seok Kim, Thomas Childs, Tammaryn Lashley, Jemima J. Burden, Michael Sasner, Carlo Sala Frigerio, Steffen Jung, Soyon Hong

**Affiliations:** 1 UK Dementia Research Institute, Institute of Neurology, University College London, London, UK; 2 VIB-KU Leuven Center for Brain and Disease Research, Leuven, Belgium; 3 Leuven Brain Institute, KU Leuven, Leuven, Belgium; 4 The Jackson Laboratory, Bar Harbor, ME USA; 5 The Queen Square Brain Bank for Neurological Disorders, Department of Clinical and Movement Neuroscience, UCL Queen Square Institute of Neurology, London, UK; 6 Department of Clinical and Movement Neuroscience, UCL Queen Square Institute of Neurology, London, UK; 7 Department of Immunology and Regenerative Biology (IRB), Weizmann Institute of Science, Rehovot, Israel; 8 Department of Neurodegenerative diseases, UCL Queen Square Institute of Neurology, London, UK; 9 Laboratory for Molecular Cell Biology, University College London, London, UK

**Keywords:** Neuroimmunology, Alzheimer's disease, Mechanisms of disease, Molecular neuroscience

Correction to: *Nature Neuroscience* 10.1038/s41593-023-01257-z, published online 6 February 2023.

A clerical error occurred during the submission of the final accepted version of this paper, in which draft versions of the source data files were mistakenly uploaded instead of the final, cross-checked versions. As a result, discrepancies arose between the published, correct figure data points and the outdated source data files for 12 out of the 21 figures. Hence, we have now thoroughly reviewed, cross-checked, and validated all source data corrections. Source Data Figs. 1–5 and Extended Data Figs. 1, 4 have been updated. A new Supplementary Table 3, overview of all the statistical tests used per figure in this study, and new Extended Data Fig. 6, raw data, are also now available with the article. Text in the main paper and Methods, and Figs. 1–5, Extended Data Figs. 1, 4 are updated throughout the paper. A detailed discussion of the changes is available as Supplementary Information alongside this amendment.

## Supplementary information

List of changes

