# Supplementary material for: Perivascular cells induce microglial phagocytic states and synaptic engulfment via SPP1 in mouse models of Alzheimer’s disease
Source: Nat Neurosci. 2023 Feb 6;26(3):406–15. doi: 10.1038/s41593-023-01257-z (PMC9991912; doi:10.1038/s41593-023-01257-z)
Supplement: Supplementary file 1 — Reporting Summary [file 41593_2023_1257_MOESM1_ESM.pdf]

nature portfolio

Corresponding author(s): Dr Soyon Hong

Last updated by author(s): Dec 8, 2022

Reporting Summary

Nature Portfolio wishes to improve the reproducibility of the work that we publish. This form provides structure for consistency and transparency in reporting. For further information on Nature Portfolio policies, see our Editorial Policies and the Editorial Policy Checklist.

Statistics

For all statistical analyses, confirm that the following items are present in the figure legend, table legend, main text, or Methods section.

n/a	Confirmed	
☐	☒	The exact sample size (*n*) for each experimental group/condition, given as a discrete number and unit of measurement
☐	☒	A statement on whether measurements were taken from distinct samples or whether the same sample was measured repeatedly
☐	☒	The statistical test(s) used AND whether they are one- or two-sided<br>*Only common tests should be described solely by name; describe more complex techniques in the Methods section.*
☒	☐	A description of all covariates tested
☐	☒	A description of any assumptions or corrections, such as tests of normality and adjustment for multiple comparisons
☐	☒	A full description of the statistical parameters including central tendency (e.g. means) or other basic estimates (e.g. regression coefficient) AND variation (e.g. standard deviation) or associated estimates of uncertainty (e.g. confidence intervals)
☐	☒	For null hypothesis testing, the test statistic (e.g. *F*, *t*, *r*) with confidence intervals, effect sizes, degrees of freedom and *P* value noted<br>*Give P values as exact values whenever suitable.*
☒	☐	For Bayesian analysis, information on the choice of priors and Markov chain Monte Carlo settings
☒	☐	For hierarchical and complex designs, identification of the appropriate level for tests and full reporting of outcomes
☒	☐	Estimates of effect sizes (e.g. Cohen's *d*, Pearson's *r*), indicating how they were calculated

Our web collection on statistics for biologists contains articles on many of the points above.

Software and code

Policy information about availability of computer code

Data collection	CLEM: Atlas 5 software and TrakEM2 (Fiji)<br>Flow Cytometry: FACSDiva software 4.0 (BD Biosciences)<br>Microscopy: Zen Blue software Version 3.3 (Zeiss), Zen Black software Version 2.3 (Zeiss) and LAS X 5.1.0 (Leica)<br>qPCR: LightCycler 96 v1.1 (Roche)
Data analysis	Data representation: Prism 9.3.1 (GraphPad Software, Inc)<br>Image Analysis: Fiji software 1.0 (NIH) and Imaris Cell Imaging Software 9.8 (Bitplane)<br>Flow Cytometry: FlowJo 10 (Treestar)<br>Single-cell RNA sequencing: R (4.1.2); 10X Genomics CellRanger (version 6.0); Seurat (4.0.6); NicheNet (1.0.0). Reads were aligned using rnaSTAR to the GRCm38 (mm10) genome.<br>Bulk-RNA seq for RiboTag analyses: Raw reads were mapped to the genome (NCBI37/mm10) using hisat (version 0.1.6).

For manuscripts utilizing custom algorithms or software that are central to the research but not yet described in published literature, software must be made available to editors and reviewers. We strongly encourage code deposition in a community repository (e.g. GitHub). See the Nature Portfolio guidelines for submitting code & software for further information.

Data

Policy information about availability of data

All manuscripts must include a data availability statement. This statement should provide the following information, where applicable:

- Accession codes, unique identifiers, or web links for publicly available datasets
- A description of any restrictions on data availability
- For clinical datasets or third party data, please ensure that the statement adheres to our policy

The scRNA-seq data generated in this study are deposited at ArrayExpress (BioStudies, Annotare 2.0) under accession number E-MTAB-11918. The RiboTag-based RNA-seq data generated in this study are deposited at ArrayExpress (BioStudies, Annotare 2.0) under accession number E-MTAB-12515. mm10 dataset is available via NCBI (GRCm38, https://www.ncbi.nlm.nih.gov/assembly/GCF_000001635.20/). Source data are provided within this paper. Raw data used in this study and requests for resources and reagents are available from the corresponding author upon request.

Human research participants

Policy information about studies involving human research participants and Sex and Gender in Research.

Reporting on sex and gender	Information regarding sex and gender is included in Supplementary Table 1, however sex and gender were not taken into consideration when including patient samples, but availability of the tissues. Findings did not apply to only one sex or gender.
Population characteristics	Post-mortem samples of 6x AD patients (5 male, 1 female; Age at Death between 61-87y) and 6 control patients (3 male, 3 female; Age at Death between 34-86y) were analyzed. See Supplementary Table 1 for ApoE, Braak Tau, Thal phase, CERAD, CAA, SVD, Alpha-syn and TDP43 stages.
Recruitment	Samples were selected based on neuropathological examination and availability of the tissues, which is unlikely to have impacted the results and conclusions.
Ethics oversight	All tissue samples were donated with the full, informed consent. Accompanying clinical and demographic data of all cases used in this study were stored electronically in compliance with the 1998 data protection act. Ethical approval for the study was obtained from the NHS research ethics committee (NEC) and in accordance with the human tissue authority's (HTA's) code of practice and standards under license number 12198, with an approved material transfer agreement. Consent has been obtained for sharing of individual-level data.

Note that full information on the approval of the study protocol must also be provided in the manuscript.

Field-specific reporting

Please select the one below that is the best fit for your research. If you are not sure, read the appropriate sections before making your selection.

☒ Life sciences ☐ Behavioural & social sciences ☐ Ecological, evolutionary & environmental sciences

For a reference copy of the document with all sections, see nature.com/documents/nr-reporting-summary-flat.pdf

Life sciences study design

All studies must disclose on these points even when the disclosure is negative.

Sample size	Study sizes were based on comparable experiments previously published (Hong et al., 2016; Hong et al., 2014).
Data exclusions	No data were excluded for analysis.
Replication	All presented data are representative of the same experiment performed in at least 3 animals. All experiments were replicated in at least two independent experiments unless stated otherwise.
Randomization	Animals were randomly assigned to different experimental groups. The allocation of post-mortem brain samples was random.
Blinding	Experiments were blinded to the genotype of the animal as well as treatment of the animal. Analysis of flow cytometry data of SPP1-TdTomato mice was performed in an unblinded fashion, for compensation purposes regarding TdTomato signal (Figure 2). Experiments with post-mortem brain samples were performed in a blinded fashion.

Reporting for specific materials, systems and methods

We require information from authors about some types of materials, experimental systems and methods used in many studies. Here, indicate whether each material, system or method listed is relevant to your study. If you are not sure if a list item applies to your research, read the appropriate section before selecting a response.

Materials & experimental systems

n/a	Involved in the study
☐	☒ Antibodies
☒	☐ Eukaryotic cell lines
☒	☐ Palaeontology and archaeology
☐	☒ Animals and other organisms
☒	☐ Clinical data
☒	☐ Dual use research of concern

Methods

n/a	Involved in the study
☒	☐ ChIP-seq
☐	☒ Flow cytometry
☒	☐ MRI-based neuroimaging

Antibodies

Antibodies used

Rabbit anti-mouse C1q (Abcam, ab182451, Clone 4.8, 1/200)
Goat anti-mouse SPP1 (Bio-Techne, AF808, 1/50)
Rabbit anti-mouse IBA1 (Wako Chemicals, 019-19741, 1/500)
Rabbit anti-mouse GLUT1 (Merck Millipore, CBL242, 1/10000)
Rat anti-mouse CD68 (Bio-Rad, MCA1957, Clone FA-11, 1/200)
Rabbit anti-mouse P2Y12 (Anaspec, AS-55043A, 1/500)
Rat anti-mouse CD206 (Bio-rad, MCA2235, Clone MR5D3, 1/400)
Chicken anti-mouse Homer1 (Synaptic Systems, 160 006, 1/500)
Mouse anti-mouse β-Amyloid, 1-16 (Biolegend, 803001, Clone 6E10, 1/200)
Mouse anti-mouse β-Amyloid, 17-24 (Biolegend, 800702, Clone 4G8, 1/200)
Rat anti-mouse CD140a (Thermo Fisher, 14-1401-82, Clone APA5, 1/50)
Rabbit anti-mouse Bassoon (Synaptic System, 141 003, 1/200)
Rabbit anti-mouse Lyve1 (Abcam, ab14917, 1/500)
Rabbit anti-human SPP1 (Merck Millipore, HPA027541-100UL, 1/200)
Mouse anti-human MMR/CD206 (Bio-Techne, MAB25341, Clone 685645, 1/200)
NAB61(1/500 kindly provided by Virginia M-Y Lee, University of Pennsylvania)
HJ5.1 (1/500 kindly provided by John R. Cirrito, WashU in St. Louis)
Alexa Fluor® 488 AffiniPure Goat Anti-Mouse IgG (H+L) (Jackson Immunoresearch, 115-545-003, 1/400)
Alexa Fluor® 594 AffiniPure Goat Anti-Rabbit IgG (H+L) (Jackson Immunoresearch, 111-585-144, 1/400)
Alexa Fluor® 647 AffiniPure Goat Anti-Rabbit IgG (H+L) (Jackson Immunoresearch, 111-605-003, 1/400)
Alexa Fluor® 647 AffiniPure Goat Anti-Rat IgG (H+L) (Jackson Immunoresearch, 112-605-167, 1/400)
Alexa Fluor® 488 AffiniPure Goat Anti-Rat IgG (H+L) (Jackson Immunoresearch, 112-545-003, 1/400)
Goat Anti-Mouse IgG Antibody (H+L), Biotinylated (Vector Laboratories, BP-9200-50, 1/200)
Goat anti-Chicken IgY (H+L) Secondary Antibody, Alexa Fluor™ 488 (Thermo Fisher, A-11039, 1/400)
Goat anti-Mouse IgG2a Cross-Adsorbed Secondary Antibody, Alexa Fluor™ 647 (Thermo Fisher, A-21241, 1/400)
Goat anti-Rat IgG (H+L) Cross-Adsorbed Secondary Antibody, Alexa Fluor™ 594 (Thermo Fisher, A-11007, 1/400)
Donkey anti-Goat IgG (H+L) Cross-Adsorbed Secondary Antibody, Alexa Fluor™ 647 (Thermo Fisher, A-21447, 1/400)
Donkey anti-Rat IgG (H+L) Highly Cross-Adsorbed Secondary Antibody, Alexa Fluor™ 488 (Thermo Fisher, A-21208, 1/400)
Donkey anti-Rabbit IgG (H+L) Highly Cross-Adsorbed Secondary Antibody, Alexa Fluor™ 594 (Thermo Fisher, A-21207, 1/400)
Goat anti-Human IgG (H+L) Cross-Adsorbed Secondary Antibody, Alexa Fluor™ 568 (Thermo Fisher, A-21090, 1/1000)
BUV395 CD45 (BD Biosciences, 564279, 1/400)
Pe-Cy7 CD11b (BD Biosciences, 552850, 1/400)
BV421 CX3CR1 (Biolegend, 149023, 1/400)
PE CD140a (Miltenyi, 130-102-473, 1/50)
APC CD206 (Biolegend, 141708, 1/400)
BV711 CD321 (BD Biosciences, 745405, 1/200)
FITC CD29 (Biolegend; 102205, 1/200)
Fixable Viability Dye eFluor™ 780 (eBioscience, 65-0865-18, 1/1000)
Monoclonal Anti-HA antibody (Sigma-Aldrich, H9658, Clone HA-7, 10 μg)
Monoclonal IgG1 antibody (Merck, Cat# PP100, 10 μg)

Validation

Each antibody was validated for the species (mouse or human) and application (immunohistochemistry, flow cytometry, immunoprecipitation) by the correspondent manufacturer. The validation studies can be found on the manufacturer's website.The usage was described in full detail the methods section of the manuscript.

Animals and other research organisms

Policy information about studies involving animals; ARRIVE guidelines recommended for reporting animal research, and Sex and Gender in Research

Laboratory animals

Animals used in this study: C57BL/6J (WT) Stock no. 000664, Age 3-15 months or neonates P0, male and female; Spp1KO/KO (B6.129S6(Cg) Stock no. 4936, Age 3-6 months or neonates P0, male; CX3CR-1GFP (B6.129P2(Cg)-Cx3cr1tm1Litt/J; Stock no. 5582, Age 3 months, male; Spp1tm1(tdTomato)Msasn mouse allele generated by Jax (Michael Sasner), Age 3 months, male and female;

	AppNL-F/NL-F mice (Saito T et al., 2014 and available via Riken, RBRC06343), age 6-15 months, male. AppNL-F/NL-F mice were crossed to Spp1KO/KO mice, age 6 months, male. All animals were housed under temperature-controlled pathogen-free conditions (22 degrees celcius) with 12 h light/dark cycle with and libitum supply of food and water. Cx3cr1ccre:Sall1ncre:R26-LSL-tdTom: Rpl22HA and Cx3cr1ccre:Lyve1ncre: R26-LSL-tdTom: Rpl22HA homozygous animals, age 3 months, male, were maintained in specific pathogen-free (SPF) conditions. Both female and male were used in this study
Wild animals	No wild animals were used in this study
Reporting on sex	Both female and male mice were used in this study. Adult mice were considered 6-12 weeks.
Field-collected samples	No field collected samples were used in this study
Ethics oversight	Experiments were performed in accordance with the UK Animal (Scientific Procedures) Act, 1986 and following local ethical advice. For the development of SPP1-TdTomato mice, all animal work was approved by the Jackson Laboratory Animal Care and Use Committee and adhered to the standards of Guide for the Care and Use of Laboratory Animals set forth by the NIH. Split-Cre animals were handled according to protocols approved by the Weizmann Institute Animal Care Committee as per international guidelines.

Note that full information on the approval of the study protocol must also be provided in the manuscript.

Flow Cytometry

Plots

Confirm that:

☒ The axis labels state the marker and fluorochrome used (e.g. CD4-FITC).

☒ The axis scales are clearly visible. Include numbers along axes only for bottom left plot of group (a 'group' is an analysis of identical markers).

☒ All plots are contour plots with outliers or pseudocolor plots.

☒ A numerical value for number of cells or percentage (with statistics) is provided.

Methodology

Sample preparation	After transcardial perfusion with 25-30 mL of filtered PBS, brains were quickly isolated from skull and hippocampus was dissected on ice using chilled instruments. Next, single cell suspension was prepared using the Adult Brain Dissociation kit from Miltenyi Biotec (Bergisch Gladbach, Germany), according manufacturer's instructions and (Frigerio C et al., 2019). Briefly, chopped tissue was incubated for 30 min in a mix of buffer Z with enzymes P, A and Y prepared according to the manufacturer's instructions. Mechanical dissociation steps were performed at 10 min intervals, first with 5 mL pipettes, then with fire-polished glass Pasteur pipettes, and lastly with P1000 tips. Afterwards, cell suspension was filtered through a 70 µM cell strainer before mixing with Debris Removal Solution. Cells were centrifuged after (300 g for 10 min) and washed with ice-cold FACS buffer (PBS, 2 % FBS, 0.78 mM EDTA). After centrifugation, cells were incubated for 30 min at 4 °C with FACS buffer containing Fc block (BD Biosciences) and primary antibody mix. Finally, right before loading the cells on the flow cytometer, cells were stained with DAPI (1:10000) for cell sorting. For flow cytometry, Fixable Viability Dye eFluor 780 was used instead of DAPI.
Instrument	BD Biosciences LSR-Fortessa X20 or AriaII for sort
Software	FlowJo 10 and FACS diva 4.0 (Treestar)
Cell population abundance	Single-cell suspensions were incubated with DAPI or Fixable Viability Dye eFluor 780 to exclude dead cells. Positive populations were gated based on negative control staining. In general, populations are given as a percentage of live, CD11B+ CD45+ cells.
Gating strategy	Cell were initially gated on FSC-A and SSC-A, and doublets were excluded. Dead cells were excluded using DAPI or Fixable Viability Dye eFluor 780. For perivascular macrophage staining, cells were gated on DAPI- CD45+ CD11B+ CX3CR1+ CD206+, and for microglia DAPI- CD45int CD11B+ CX3CR1hi CD206-. Perivascular fibroblasts and oligodendrocytes were considered CD11B- CX3CR1- PDGFRA+

☒ Tick this box to confirm that a figure exemplifying the gating strategy is provided in the Supplementary Information.
