# Supplementary material for: Author Correction: Perivascular cells induce microglial phagocytic states and synaptic engulfment via SPP1 in mouse models of Alzheimer’s disease
Source: Nat Neurosci. 2026 Feb 17;29(3):759. doi: 10.1038/s41593-025-02197-6 (PMC12971480; doi:10.1038/s41593-025-02197-6)
Supplement: Supplementary file 1 — List of changes [file 41593_2025_2197_MOESM1_ESM.pdf]

nature neuroscience

Supplementary information

Author Correction: Perivascular cells induce microglial phagocytic states and synaptic engulfment via SPP1 in mouse models of Alzheimer's disease

In the format provided by the authors and unedited

Supplementary Information to Author Correction: Perivascular cells induce microglial phagocytic states and synaptic engulfment via SPP1 in mouse models of Alzheimer's disease

Sebastiaan De Schepper, Judy Z. Ge, Annerieke Sierksma, Gerard Crowley, Laís S. S. Ferreira, Dylan Garceau, Christina E. Toomey, Dimitra Sokolova, Javier Rueda-Carrasco, Sun-Hye Shin, Jung-Seok Kim, Thomas Childs, Tammaryn Lashley, Jemima J. Burden, Michael Sasner, Carlo Sala Frigerio, Steffen Jung & Soyon Hong

Correction to: *Nature Neuroscience* https://doi.org/10.1038/s41593-023-01257-z, published online 6 February 2023.

Main article

1. Updated statistical tests:

We revised the statistical analyses for Fig. 4h and Ext. Data Fig. 5c, where we replaced two-way ANOVA with multiple unpaired t-tests with Bonferroni-Dunn correction. We also refined statistical tests to enhance accuracy and corrected instances where post-hoc results were reported despite non-significant ANOVA interactions. These updates are detailed below. For transparency, we added a new table, Supplementary Table 3, which details the statistical tests used in the corrected paper and have expanded the *Statistics* subsection of the *Material and Methods*. Notably, these updated and corrected analyses led to results consistent with our original interpretation, and the overall conclusions of the paper remain unchanged.

Corrected stats for Figure 4h, Extended Data Figure 5c: Synapse numbers, defined by colocalization of Bassoon and Homer1-immunoreactive puncta, were assessed in two models of Aβ challenge: transgenic *App*$^{NL\text{-}F/NL\text{-}F}$ vs. WT (Fig. 4h) and acute Aβ oligomer-injected WT vs. vehicle-injected WT (Ext. Data Fig. 5c). These experiments were conducted first in *Spp1*$^{WT/WT}$ background, and then in *Spp1*$^{KO/KO}$ background, using AiryScan confocal microscopy and Imaris 3D analysis with experimenters blinded to genotype/condition. Each Aβ-challenged animal was paired with a control (age- and sex-matched), and synapse counts were normalized to their respective control groups. Consistent with previous publications, synapse loss was observed in Aβ models in *Spp1*$^{WT/WT}$ background. We then compared the effects of Aβ in *Spp1*$^{KO/KO}$ background. Given the paired experimental design and control normalization, data points are not independent, rendering two-way ANOVA an inappropriate statistical test for these experiments. We therefore applied the multiple unpaired t-tests for each comparison, using the Bonferroni-Dunn correction to account for multiple testing and controlling for type I error.

Figure 4h:

Published: | **Corrected:**

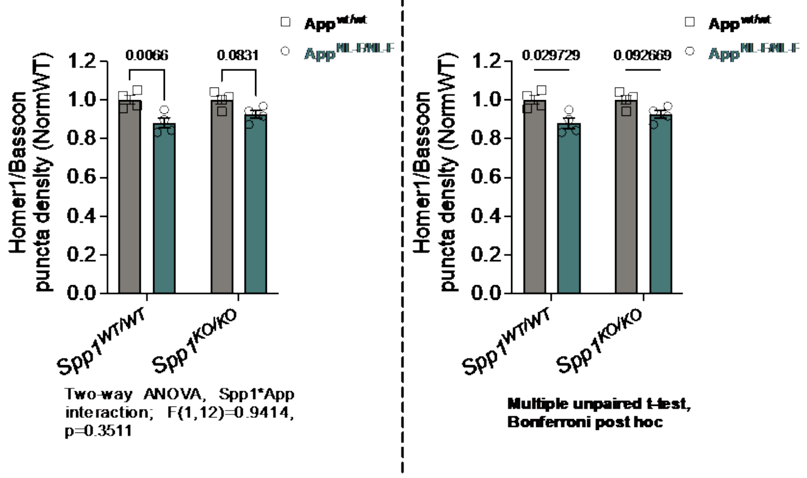

Corrected legend: (**h**) Quantification of Homer1/Bassoon colocalization density normalized to WT or *Spp1*$^{KO/KO}$ accordingly.1 datapoint represents the average of 1 animal (3-5 ROIs per animal) with a total of n=4 animals per genotype. Statistics done per mouse. *P*< 0.029729 (WT) and 0.092669 (*Spp1*$^{KO/KO}$) from multiple unpaired t-test, Bonferroni's multiple comparison test. Data are shown as Mean ± SEM.

Extended Data Figure 5c:

Published: | **Corrected:**

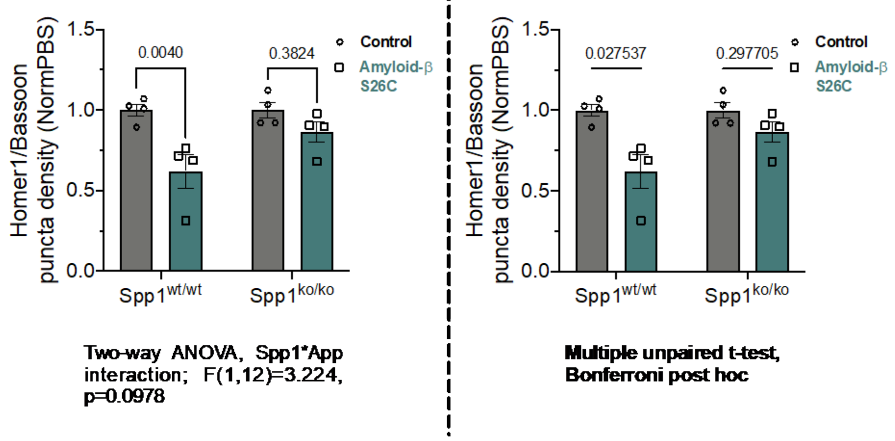

Corrected legend: (**c**) Quantification of Homer1/Bassoon colocalization density, represented as S26C oAβ-injected mice normalized to PBS injected mice. 1 datapoint represents the average of 1 animal (3-5 ROIs per animal, 40 cells per ROI) with a total of n=4 mice per genotype. Statistics done per mouse. *P* Values from multiple unpaired t-tests, Bonferroni's multiple comparison test. Data are shown as Mean ± SEM.

Refined stats for Figure 1: We originally applied conservative Mann-Whitney U test (two-tailed), which is appropriate given the small sample size. However, because the residuals of the data fit a normal distribution, we acknowledge that the unpaired Student's t-test (two-tailed) is more appropriate. The revised analyses lead to the following:

- Fig. 1d (C1q protein) published ***p=0.0229** vs. revised ****p=0.0072**.
- Fig. 1f (SPP1 protein) published ***p=0.0480** vs. revised ****p=0.0094**.
- Fig. 1g (*Spp1* mRNA using smFISH) published ***p=0.0230** vs. revised ****p=0.001**.
- Fig. 1h (*Spp1* mRNA using qPCR) published ***p=0.0317** vs. revised ***p=0.0499**. **Welsh's correction applied due to unequal variances.*

Modified legends:
Fig. 1 (d). Quantification of C1q puncta in 6 mo WT and *App*$^{NL-F}$ CA1 hippocampus. 1 datapoint represents 1 ROI per mouse from n=9 WT mice and n=7 *App*$^{NL-F}$ mice examined over 2 independent experiments. Average amount of cells per datapoint is 30-40. *P* Values from Two-tailed unpaired Student's t-test.
Fig. 1 (f). 1 datapoint represents 1 ROI per mouse, with total of n=3 mice examined over 1 independent experiment. *P* Values from Two-tailed unpaired Student's t-test. Scale bar represents 2 μm.
Fig. 1 (g-h). 1 datapoint represents 1 individual value per mouse, with total of n=5-6 mice (per genotype) **(g)** or 4-5 mice (per genotype) **(h)** examined over 2 independent experiments. *P* Values from Two-tailed unpaired Student's t-test with Welch's correction for (h).

Refined stats for Figure 5f: We originally applied 1-way ANOVA considering genotype as a single variable, assumptions made a priori and the experimental design, where we placed four genotypes on the same slide per experiment. However, we acknowledge that there is a strong rationale for treating *App* and *Spp1* genotypes as two independent variables given the overall goal of our paper, without violating the assumptions made a priori and the experimental design. Therefore, we applied 2-way ANOVA, with Bonferroni correction for pair-wise comparisons in case of significant interactions.

Figure 5f: CD29

Published:

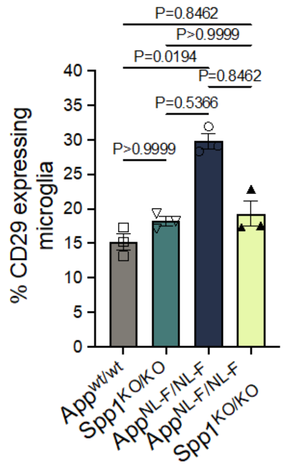

1 dot = 1 mouse. Kruskal–Wallis test. p=0.0064

Corrected:

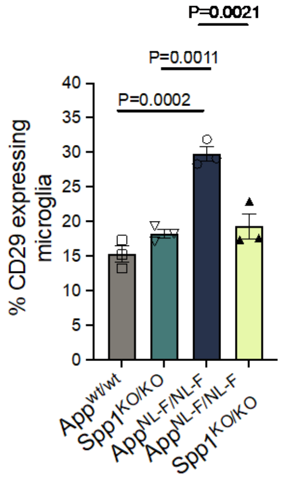

Two-way ANOVA, Spp1*App interaction; F(1,8)=28.92, p=0.0007. Bonferroni post hoc

Figure 5f: CD321

Published:

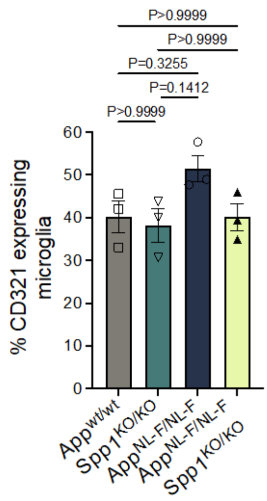

1 dot = 1 mouse. Kruskal–Wallis test. p=0.0788

Corrected:

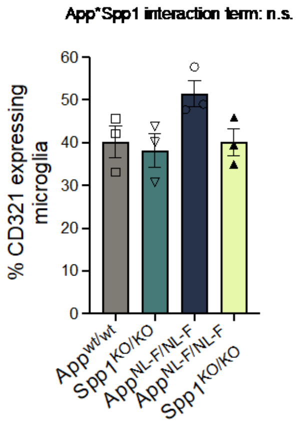

Two-way ANOVA, Spp1*App interaction; F(1,8)=1.755, p=0.2218.

Corrected legend: **Fig. 5 (f).** Quantification of NicheNet hits CD29 (*Itgb1)* and CD321 (*F11r)* on microglia (CX3CR1^{high}CD45^{+}CD11b^{+} CD206^{-}) isolated from hippocampal homogenates of 6 mo WT, *Spp1$^{KO/KO}$*, *App*$^{NL-F}$ and *App*$^{NL-F}$.*Spp1$^{KO/KO}$* animals. 1 datapoint represents 1 individual mouse (microglia) pooled from n=3 mice from 1 experiment. Statistics done per mouse. *P* Values from Two way ANOVA, Bonferroni's multiple comparisons test (CD29).

Refined stats for Extended Data Figure 1ab: We originally used the Mann-Whitney U test to test SPP1 protein staining difference between WT and *App$^{NL-F/NL-F}$* mice in hippocampus (CA1) (Ext. Data Fig. 1a) and, separately, in cerebellum (CRB; Ext. Data Fig. 1b). We have now applied a linear mixed-effect model to compare across the two brain regions. This model accounts for inter-mouse variability by including 'mouse' as a nested variable, while testing for the effects of region, genotype, and their interaction. After identifying a significant interaction effect, we used a multivariate t-distribution post-hoc test to evaluate the impact of genotype on SPP1 expression in both regions (see Supplementary Table 3). The refined stats change

the p values from ***p=0.0499 (CA1)** to now to ***p=0.0489 (CA1)**, and **p>0.9999 (NS) (CRB) to p=0.9195 (NS) (CRB)**.

Corrected legend: 1 datapoint represents 1 ROI per mouse hippocampus, with total of 8 (CA1, a) and 7 (CRB, b) mice per genotype examined over 2 independent experiments. *P* Values from linear mixed-effect model with multivariate t-distribution post-hoc test. Data are shown as Mean ± SEM.

2. Update from per-microglia to per-mouse representation:

In our original publication, we chose to represent data per-microglia because we observed that variability in microglia-synapse engulfment was often greater within a brain region of a given mouse than between different mice. However, we acknowledge that representing the data per-microglia does not account for inter-mouse variability. Therefore, we corrected all per-microglia figures (Figures 1b, 3g, 3h, 4b, 4d, 4f and 5e) to per-mouse data and performed appropriate statistical tests. These corrections do not alter the major data interpretations in any of the figures, except for Fig. 4d (*Grn*), where the effect now represents a trend.

Figure 1b:

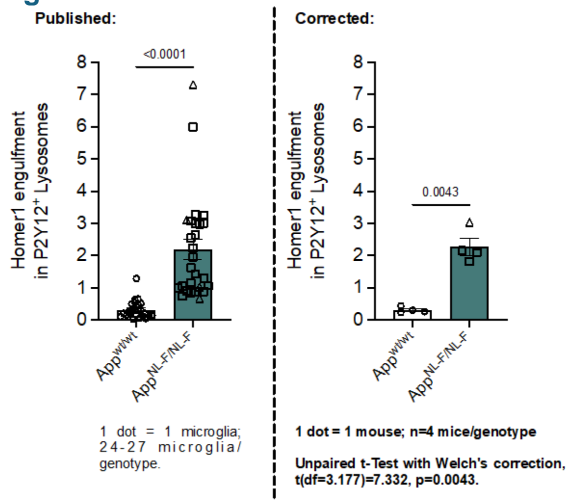

Corrected legend: **Fig. 1 (b).** Quantification of Homer1 engulfment in 6 mo WT and $App^{NL\text{-}F}$ P2Y12⁺ microglia. 1 datapoint represents average of 1 mouse, from n=4 animals per genotype examined over 2 independent experiments. *P* Values from two-tailed unpaired Student's t-test with Welch's correction.

Figure 3g:

Published:

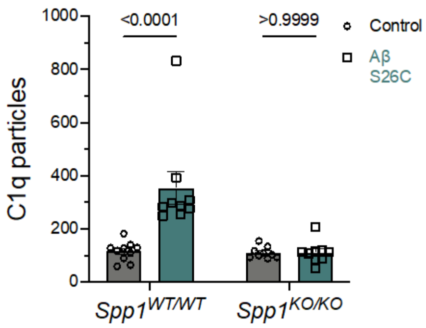

1 dot = 1 ROI;
3-4 ROI/ genotype.

Corrected:

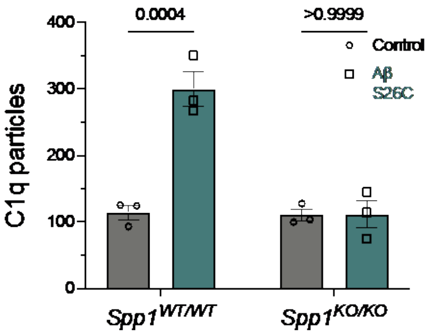

1 dot = 1 mouse; n=3 mice/genotype

Two-way ANOVA, Spp1 genotype*treatment interaction; F(1,8)=27.29, p=0.0008; Bonferroni post hoc.

Corrected legend: **Fig. 3 (g).** Quantification of C1q particles (puncta) in WT or *Spp1*$^{KO/KO}$ mice treated with either PBS or oAβ, as in **(c,e).** 1 datapoint represents average of 1 mouse, calculated from 3-4 ROIs per mouse from n=3 mice, examined over 2 independent experiments. *P* Values from Two-way Anova, Bonferroni's multiple comparison test.

Figure 3h:

Published:

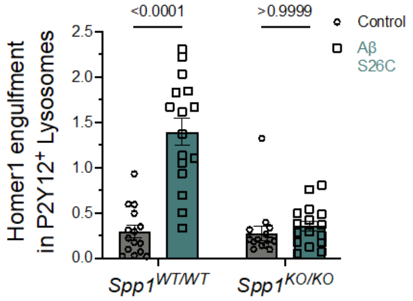

1 dot = 1 microglia; 5-11 microglia/ genotype.

Corrected:

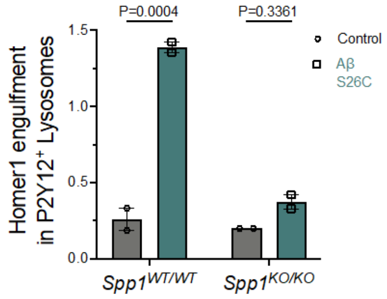

1 dot = 1 mouse; n=2 mice/genotype.

Two-way ANOVA, Spp1 genotype*treatment interaction; F(1,4)=107.4, p=0.0005; Bonferroni post hoc.

Corrected legend: **Fig. 3 (h).** Quantification of Homer1 engulfment in WT or *Spp1*$^{KO/KO}$ P2Y12^{+} microglia, ICV treated with either PBS or oAβ, as in **(d,f).** 1 datapoint represents average of 1 mouse, calculated from 5-11 individual P2Y12^{+} microglia per mouse from n=2 animals examined over 2 independent experiments. *P* Values from Two-way Anova, Bonferroni's multiple comparison test. Data are shown as mean ± SEM.

Figure 4b,d:

Figure 4b:

Published:

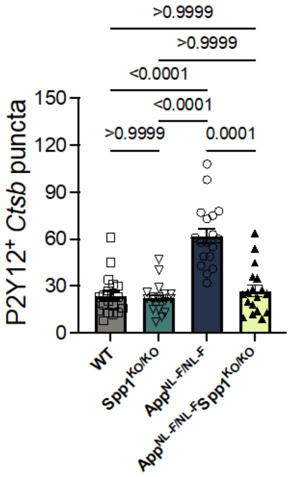

1 dot = 1 microglia. 6 microglia/ genotype.

Corrected:

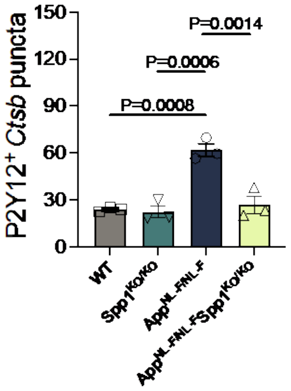

1 dot = 1 mouse; n=3 mice/genotype

Two-way ANOVA, Spp1*App interaction; F(1,8)=18.33, p=0.0027; Bonferroni post hoc.

Figure 4d:

Published:

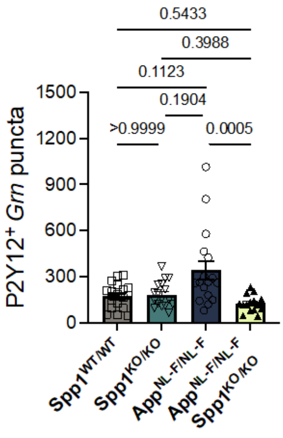

1 dot = 1 microglia. 3-6 microglia/ genotype.

Corrected:

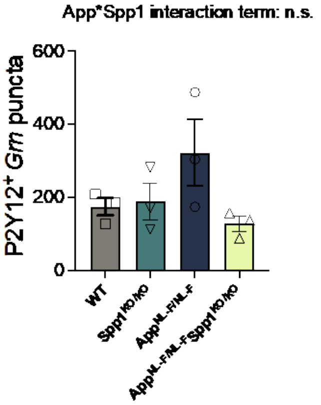

1 dot = 1 mouse; n=3 mice/genotype

Two-way ANOVA, Spp1*App interaction; F (1,8)=3.863, p=0.0849

Corrected legend: **Fig. 4 (b,d).** Quantification of *Ctsb* **(b)** and *Grn* **(d)** mRNA levels expression within P2Y12+ microglia. 1 datapoint represents average of 1 mouse, calculated from 6 individual P2Y12+ microglia per mouse **(b)** and 3-6 microglia **(d)** from n=3 mice per genotype examined over 2 independent experiments. *P* Values from Two-way ANOVA, Bonferroni's multiple comparison test for (b).

Figure 4f:

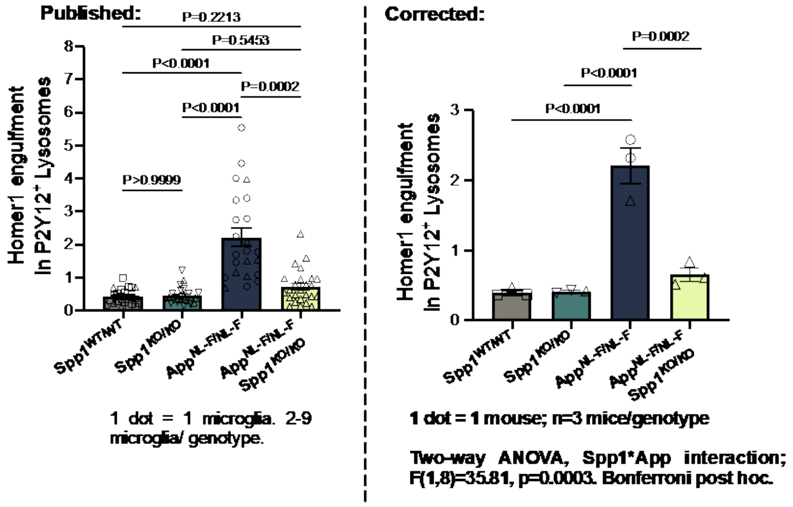

Corrected legend: **Fig. 4 (f).** Quantification of Homer1 engulfment ratio in P2Y12^{+} microglia of WT versus *Spp1*$^{KO/KO}$ versus *App*$^{NL\text{-}F}$ versus *App*$^{NL\text{-}F}$.*Spp1*$^{KO/KO}$ mice. 1 datapoint represents average of 1 mouse, calculated from 7-9 individual P2Y12^{+} microglia per mouse from n=3 mice examined over 2 independent experiments. Statistics done per mouse. *P* Values from Two-way ANOVA, Bonferroni's multiple comparison test.

Figure 5e:

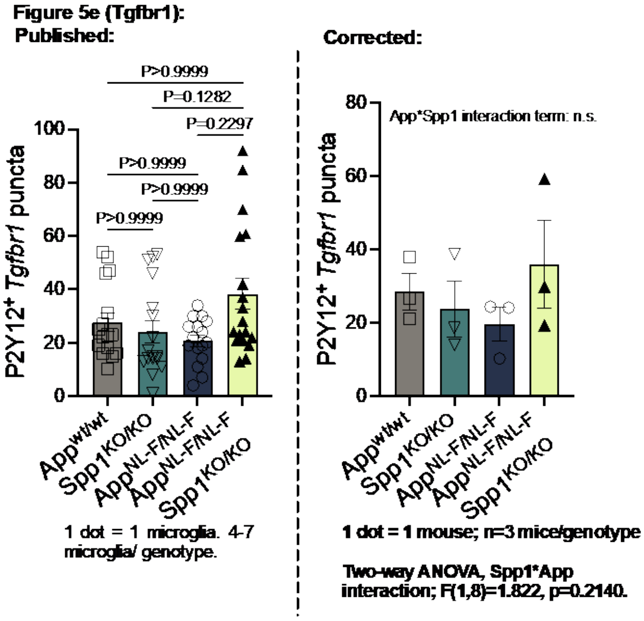

Figure 5e (Itgb5):

Published:

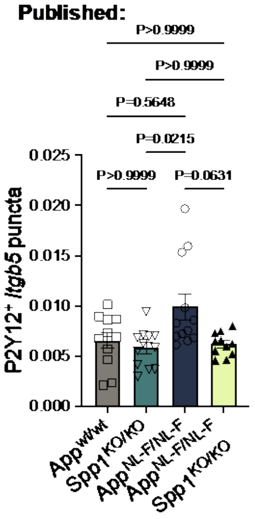

1 dot = 1 microglia. 3-4 microglia/ genotype.

Corrected:

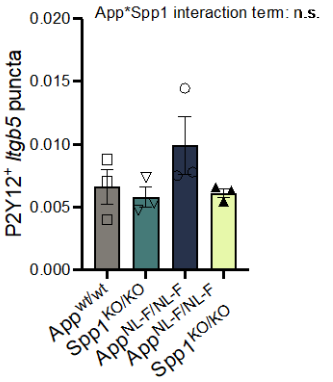

1 dot = 1 mouse; n=3 mice/genotype

Two-way ANOVA, Spp1*App interaction; F(1,8)=1.040, p=0.3378.

Corrected legend: **Fig. 5 (e).** Quantification of *Tgfbr1* and *Itgb5* mRNA levels expressed by P2Y12^{+} microglia assessed by smFISH-IHC in 6 mo WT, *Spp1$^{KO/KO}$, App*$^{NL-F}$ and *App*$^{NL-F}$.*Spp1$^{KO/KO}$* SLM. 1 datapoint represents average of 1 mouse, calculated from 3-7 individual P2Y12^{+} microglia per mouse from n=3 animals examined over 1 independent experiment. *P* Values from Two-way ANOVA.

3. Text

The following sentences have been updated in the main text.

New text is on the bottom, old text is on the top

(Abstract) Perivascular SPP1 is required for microglia to engulf synapses and upregulate phagocytic markers including *C1qa*, *Grn* and *Ctsb* in presence of amyloid-β oligomers.

(Abstract) Perivascular SPP1 is required for microglia to engulf synapses and upregulate phagocytic markers including *C1qa* and *Ctsb* in presence of amyloid-β oligomers.

Further, only approximately 6 % of total live TdT^{+} cells were CD140a^{+} (gene product of *Pdgfra*), in contrast to 23.9 % for CD206^{+}, highlighting PVM as a predominant cellular source of SPP1 (Fig. 2e-f)

Further, only approximately 12 % of total live TdT^{+} cells were CD140a^{+} (gene product of *Pdgfra*), in contrast to 20.5 % for CD206^{+}, highlighting PVM as a predominant cellular source of SPP1 (Fig. 2e-f).

Using smFISH-HC and 3D reconstruction, we observed upregulation of *Grn* and *Ctsb*, encoding for progranulin and Cathepsin B respectively, key components of the endolysosomal processing machinery in P2Y12^{+} microglia (Fig. 4a-d). However, in the *App*$^{NL-F}$.*Spp1$^{KO/KO}$* hippocampus, we found significantly decreased levels of *Grn and Ctsb* mRNA expression by P2Y12^{+} microglia (Fig. 4a-d).

Using smFISH-HC and 3D reconstruction, we observed an upregulation trend for *Grn* and a significant increase for *Ctsb*, encoding for progranulin and Cathepsin B respectively, key components of the endolysosomal processing machinery in P2Y12^{+} microglia (Fig. 4a-d).

However, in the $App^{NL\text{-}F}$.$Spp1^{KO/KO}$ hippocampus, we found significantly decreased levels of *Ctsb* mRNA expression by $P2Y12^{+}$ microglia, with *Grn* showing a downward trend (Fig. 4a-d).

Finally, to determine the consequence of *Spp1* deficiency on synapse numbers in 6 mo $App^{NL\text{-}F}$ animals, we used super-resolution microscopy to analyze pre- and post-synaptic markers (Bassoon and Homer1, respectively) in the hippocampal CA1 stratum radiatum of 6 mo $App^{NL\text{-}F}$ mice (Fig. 4g,h).

Finally, to determine the consequence of *Spp1* deficiency on synapse numbers in 6 mo $App^{NL\text{-}F}$ animals, we used super-resolution microscopy to analyze pre- and post-synaptic markers (Bassoon and Homer1, respectively) in the hippocampal CA1 SLM of 6 mo $App^{NL\text{-}F}$ mice (Fig. 4g,h).

We further validated these results via smFISH-IHC; as suggested by NicheNet, *Tgfbr1* and *Itgb5* expression levels were dysregulated in microglia of $App^{NL\text{-}F}$.$Spp1^{KO/KO}$ versus $App^{NL\text{-}F}$ mice (Fig. 5e; Extended Data Fig. 6e,f). In addition, we confirmed decreased expression of CD29 (*Itgb1*) and CD321 (*F11r*) in hippocampal microglia of $App^{NL\text{-}F}$.$Spp1^{KO/KO}$ compared to $App^{NL\text{-}F}$ animals by flow cytometry (Fig. 5f,g; Extended Data Fig. 6f).

We further validated these results via smFISH-IHC; as suggested by NicheNet, *Tgfbr1* and *Itgb5* expression levels showed a trend towards dysregulation in microglia of $App^{NL\text{-}F}$.$Spp1^{KO/KO}$ versus $App^{NL\text{-}F}$ mice (Fig. 5e; Extended Data Fig. 6e,f). In addition, we confirmed decreased expression of CD29 (*Itgb1*) and a trending reduction in CD321 (*F11r*) in hippocampal microglia of $App^{NL\text{-}F}$.$Spp1^{KO/KO}$ compared to $App^{NL\text{-}F}$ animals by flow cytometry (Fig. 5f,g; Extended Data Fig. 6f).

Indeed, in the absence of *Spp1*, microglia fail to upregulate key phagocytic and AD-relevant genes such as progranulin and *Ctsb* in 6 mo $App^{NL\text{-}F}$ mice. Of note, phagocytosis induced by microglial progranulin as well as complement proteins including C3 has been considered protective against Aβ plaque load and neuronal loss in plaque-rich mouse models of AD.

Indeed, in the absence of *Spp1*, microglia fail to upregulate key phagocytic and AD-relevant *Ctsb* in 6 mo $App^{NL\text{-}F}$ mice. Of note, phagocytosis induced by complement proteins including C3 has been considered protective against Aβ plaque load and neuronal loss in plaque-rich mouse models of AD.

In contrast to decreased *Tgfbr2,* we observed increased *Tgfbr1* signaling in microglia of $App^{NL\text{-}F}$.$Spp1^{KO/KO}$ versus $App^{NL\text{-}F}$ animals.

In contrast to decreased *Tgfbr2,* we observed trend towards increased *Tgfbr1* signaling in microglia of $App^{NL\text{-}F}$.$Spp1^{KO/KO}$ versus $App^{NL\text{-}F}$ animals.

4. <u>Figure legends</u>

Additional updates to figure legends:

New figure legend is on the bottom, old figure legend is on the top

Fig. 2 (f). *P* Values from one way ANOVA, Kruskal-Wallis test.

Fig. 2 (f). *P* Values from one-way ANOVA, Bonferroni's multiple comparison test.

Fig. 3 (b). *P* Values from Two-way ANOVA.

Fig. 3 (b). *P* Values from Two-way ANOVA, Bonferroni's multiple comparison test.

Extended Data Fig. 4 (d): Primary microglial cultures have been prepared from n=6-8 neonates per genotype, examined over of 2 independent experiments.

Extended Data Fig. 4 (d): Primary microglial cultures have been prepared from n=5-8 neonates per genotype, examined over of 2 independent experiments.`

Extended Data Fig. 4 (e): *P* Values from two way ANOVA, Bonferroni's multiple comparison test. Data are shown as Mean ± SEM.

Extended Data Fig. 4 (e): *P* Values from two-way ANOVA, Bonferroni's multiple comparison test. Overall treatment effect $F(1,24)=43.29$, $p<0.0001$. Data are shown as Mean ± SEM.

Extended Data Fig. 5: (a) Scheme illustrating intracerebroventricular (ICV) injection

Extended Data Fig. 5: (a) Scheme illustrating tail vein injection

5. Figures

Additional updates (statistical annotations and graph data) to figures to reflect the statistical corrections:

New graph elements are on the bottom, old graph elements are on the top

Published: **Fig. 2 (e).** 6.1 ± 2.1 and 23.9 ± 2.9

Corrected: **Fig. 2 (e).** 11.9 ± 2.0 and 20.5 ± 2.0

Published: **Fig. 3 (b).** p=0.0004 (*Spp1*$^{WT/WT}$, 18h); p=0.9958 (*Spp1*$^{WT/WT}$, 72h)

Corrected: **Fig. 3 (b).** p=0.0011 (*Spp1*$^{WT/WT}$, 18h); p>0.9999 (*Spp1*$^{WT/WT}$, 72h)

Published: **Extended Data Fig. 4e:** p=0.0003 and p=0.0008

Corrected: **Extended Data Fig. 4e:** Treatment effect p<0.0001

Methods

a) We expanded the Statistics section in the Materials and Methods for improved clarity and transparency.

Statistics: All statistical analyses were performed in Prism (GraphPad Software, Version 9.3.1) or R (v.4.3.2) and a complete overview is provided in Supplemental Table 3. Outliers were identified and removed from the dataset using Graphpad Prism (ROUT, Q=1%). Normal distribution and equality of variance of the residuals were tested using the Shapiro-Wilk normality test and the F-test, Spearman's test or Bartlett's test, using significance level $\alpha = 0.05$. To stabilize variances on heteroscedastic residuals, we performed log10 transformations on the data for Fig. 4d, 4f and Ext. Data Fig. 4e before fitting the two-way ANOVA model (linear data was used for plotting to facilitate interpretation of the outcome). Two groups were compared using two-tailed unpaired Student's t-test, two-tailed Mann–Whitney U tests or Welch's t-test, depending on the data structure. To compare more than two groups (PVMs,

microglia and PVFs), one-way ANOVA with Bonferroni's multiple comparison post hoc test was used. Two-way ANOVA with Bonferroni's multiple comparison test was used to assess the impact of Aβ exposure (App^{NL-F} genotype or oAβ injection) and Spp1 genotype ($Spp1^{WT/WT}$, $Spp1^{KO/KO}$), and the impact of recombinant SPP1 on both $Spp1^{WT/WT}$ and $Spp1^{KO/KO}$ mice. Due to the technical approach in synapse analysis studies, the analyses were performed in pairs, $App^{NL-F/NL-F}$ mice were normalized to WT mice and $App^{NL-F/NL-F}$·$Spp1^{KO/KO}$ mice were normalized to $Spp1^{KO/KO}$ mice, or oAβ injected mice were paired with control injected mice. As such, we performed two separate two-tailed unpaired t-tests between the sets, to which a Bonferroni correction for multiple comparisons was applied to reduce the likelihood of a type I error. For brain region (hippocampus vs. cerebellum) vs. genotype (WT vs. App^{NL-F}) analysis, we used a linear mixed-effect model with multivariate t-distribution post-hoc, using mouse as a nested variable to account for inter-mouse variability [R packages lme4 (v.1.1-35.2), emmeans (v.1.10.0), lmerTest (v. 3.1-3)]. All data is presented as mean +/- SEM, statistical significance was set at α = 0.05. Data in graphs are presented as mean ± s.e.m. Statistical methods were not used to predetermine study sizes but were based on similar experiments previously published. Experiments were blinded to the genotype of the animal as well as the treatment of the animal. Experiments involving human sections were blinded to the demographics of the patients. Analysis of flow cytometry data of $Spp1^{TdT}$ mice was performed in an unblinded fashion, for compensation purposes.

b) We added previously omitted information regarding antibodies in the Materials and Methods section. Additions are in red font below:

Antibodies: PE CD140a (Miltenyi, 130-102-502; 1/50), APC CD140a (Miltenyi, 130-102-473; 1/50), APC CD206 (Biolegend, 141708; 1/400), FITC CD206 (Biolegend, 141704; 1/400), FITC CD29 (Biolegend, 102205; 1/200) and BV711 CD321 (BD Biosciences, 745405; 1/200) were used.

c) We corrected the region of interest in the methods section, which was erroneously referred to as CA1 stratum radiatum. The correct region is CA1 stratum lacunosum moleculare:

Super-resolution imaging and synapse analysis: Three regions of interest of 1.15 μm were acquired in the center of the hippocampal CA1 stratum lacunosum moleculare (SLM) for each brain section.

Acknowledgements and Author list:

We have added Dr. Annerieke Sierksma as a co-author for her extensive statistical input, and acknowledged two statisticians, Daan Moechars, MSc, and Prof. Saiful Islam, for their critical input regarding the most appropriate statistical tests and data analysis. Dr. Sierksma thoroughly cross-checked the results and performed independent statistical tests using R software. Dr. Sierksma and Moechars are affiliated with VIB-KU Leuven Center for Brain and Disease Research (Leuven, Belgium) and Prof. Islam is an Associate Professor in Medical Statistics at UCL Queen Square Institute of Neurology.

Supplementary Table

We have added Supplementary Table 3, which provides a detailed overview of all the updated statistical tests used in the paper.

Source Data Files

A clerical error occurred during the submission of the final accepted version of the paper, in which draft versions of the source data files were mistakenly uploaded instead of the final, cross-checked versions. As a result, discrepancies arose between the published, correct figure data points and the outdated source data files for 12 out of the 21 figures. Hence, we have now thoroughly reviewed, cross-checked, and validated all source data corrections. The corrected source data files are now updated with corrections highlighted in red.
